# The chloroplast genome sequence of bittersweet (*Solanum dulcamara*): Plastid genome structure evolution in Solanaceae

**DOI:** 10.1371/journal.pone.0196069

**Published:** 2018-04-25

**Authors:** Ali Amiryousefi, Jaakko Hyvönen, Péter Poczai

**Affiliations:** 1 Organismal Evolutionary Biology Research Program, Faculty of Biology and Environmental Sciences, Viikki Plant Science Centre, University of Helsinki, Helsinki, Finland; 2 Finnish Museum of Natural History (Botany), University of Helsinki, Helsinki, Finland; Austrian Federal Research Centre for Forests BFW, AUSTRIA

## Abstract

Bittersweet (*Solanum dulcamara*) is a native Old World member of the nightshade family. This European diploid species can be found from marshlands to high mountainous regions and it is a common weed that serves as an alternative host and source of resistance genes against plant pathogens such as late blight (*Phytophthora infestans*). We sequenced the complete chloroplast genome of bittersweet, which is 155,580 bp in length and it is characterized by a typical quadripartite structure composed of a large (85,901 bp) and small (18,449 bp) single-copy region interspersed by two identical inverted repeats (25,615 bp). It consists of 112 unique genes from which 81 are protein-coding, 27 tRNA and four rRNA genes. All bittersweet plastid genes including non-functional ones and even intergenic spacer regions are transcribed in primary plastid transcripts covering 95.22% of the genome. These are later substantially edited in a post-transcriptional phase to activate gene functions. By comparing the bittersweet plastid genome with all available Solanaceae sequences we found that gene content and synteny are highly conserved across the family. During genome comparison we have identified several annotation errors, which we have corrected in a manual curation process then we have identified the major plastid genome structural changes in Solanaceae. Interpreted in a phylogenetic context they seem to provide additional support for larger clades. The plastid genome sequence of bittersweet could help to benchmark Solanaceae plastid genome annotations and could be used as a reference for further studies. Such reliable annotations are important for gene diversity calculations, synteny map constructions and assigning partitions for phylogenetic analysis with de novo sequenced plastomes of Solanaceae.

## Introduction

The genus *Solanum* L., with approximately 1,400 species, is one of the largest genera of angiosperms, and includes many major and minor food crops such as tomato, potato, eggplant, and pepino. Bittersweet (*Solanum dulcamara* L.) is a European native diploid (2n = 2× = 24) species, which is found throughout the northern hemisphere across a wide range of habitats. It was also introduced to North America possibly for its medicinal properties [1]. It is still used as a source of various alkaloids with diuretic, diaphoretic properties to treat rheumatism and skin diseases in Asia and India [2, 3].

**Fig 1:**
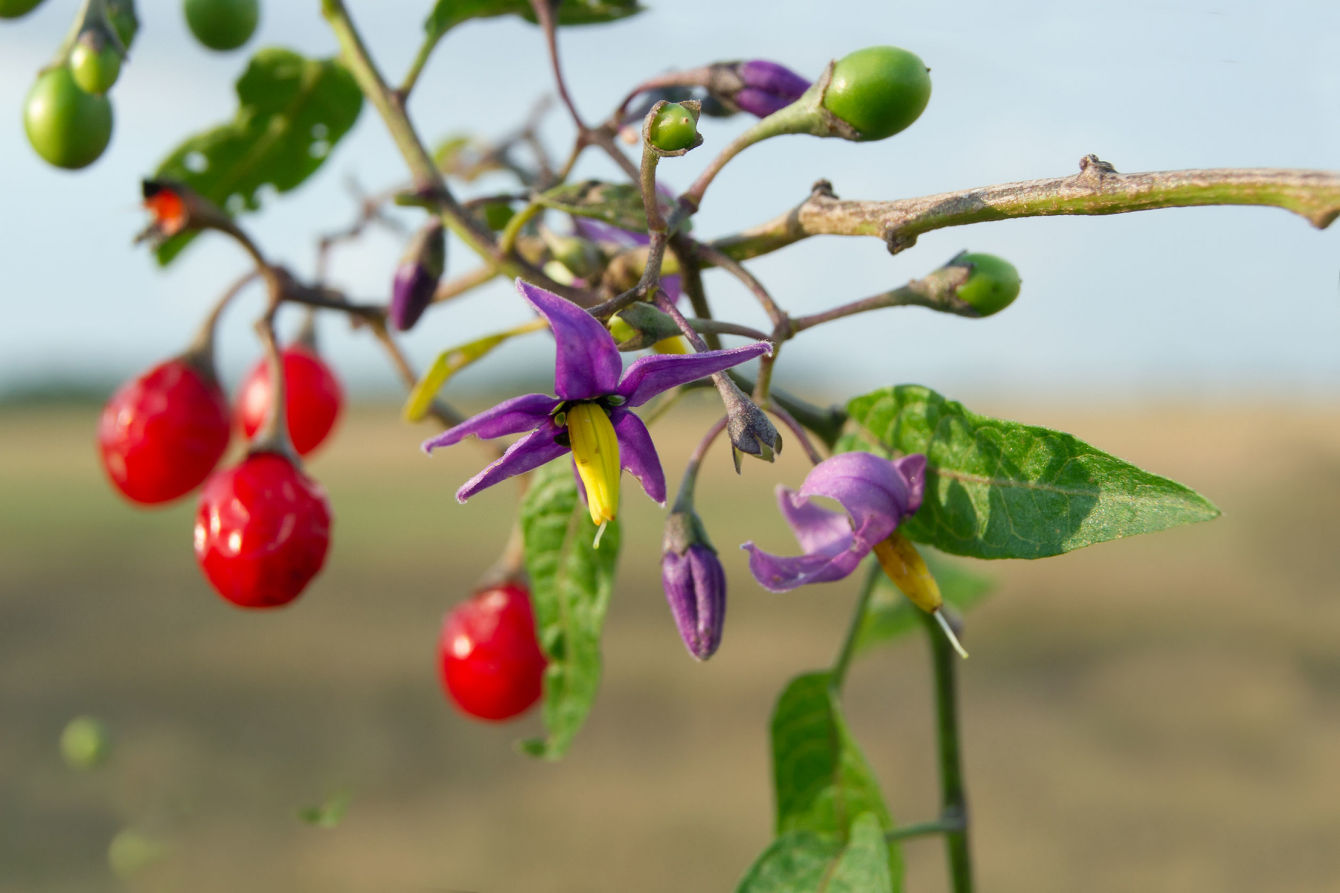
The berries and flowers of *Solanum dulcamara* L.

This semi-woody perennial vine is easy to recognize (Fig 1). However, it is a highly polymorphic and phenotypically plastic species showing extreme forms, which has led to confused taxonomy. Previous treatments placed *Solanum dulcamara* to sect. *Dulcamara* (Moench) Dumort. in subg. *Potatoe* (G.Don) D’Arcy related to potatoes (sect. *Petota* Dumort.) and tomatoes (sect. *Lycopersicum* (Tourn.) Wettst.) [4–7]. This was based on scandent habit, pinnate leaves and on the articulation of pedicels above the base [1, 4]. However, recent phylogenetic studies have shown that it belongs to the Dulcamaroid clade [8–11], which is closely related to the Morelloid clade including species of black nightshades of sect. *Solanum* (e.g. *S*. *nigrum* L. and *S*. *scabrum* Mill.).

*Solanum dulcamara* serves as a host for important plant pathogens such as those causing bacterial wilt (*Ralstonia solanacearum* (Smith 1896) Yabuuchi et al. 1996), late blight (*Phytophthora infestans* (Mont.) de Bary.) and also for some viruses [12, 13]. Late blight, is one of the most serious potato diseases worldwide [14]. However, it was shown that bittersweet has a minimal role in late blight infections since most plants are resistant and the inocula of the pathogen do not overwinter [15]. Populations of this species seem to have experienced a genetic bottleneck [16], but some allelic variation was found to be distributed among populations resulting in more structured populations at larger regional levels [17]. The differentiation of the populations could have arisen by genetic drift or even by inbreeding over a very long period. Bittersweet is mostly an outcrossing species, but its population structure might have been affected by its perennial self-compatibility [18], reducing genetic diversity within regional populations and enhancing inbreeding. This leads to high interpopulation or spatial differentiation [17]. Genetic drift, on the other hand, may not have shaped the population structure of the species recently based on the observed moderate level of diversity among populations [16, 17]. However, over a longer time scale population expansion from postglacial refugia is known to leave such traces [19].

High throughput sequencing is revolutionizing phylogenetics as it allows to obtain hundreds to thousands of markers in a cost effective way. Complete plastid genome (plastome) sequences now could be easily acquired for phylogenomic analyses with relatively low cost. Angiosperm plastid genomes exist in circular and linear forms [20] and the percentage of each form varies within plant cells [21]. They are small, typically ~ 120–150 kb in size and have a highly conserved quadripartate structure containing two inverted repeats (IRA and IRB), which separate the large and small single copy regions (LSC and SSC). The plastid genome includes 110–130 genes primarily participating in photosynthesis, transcription and translation [22]. Their conserved gene content, order and organization makes them relatively well suited for evolutionary studies since gene losses, structural rearrangements, pseudogenes or additional mutation events could be characteristic for some lineages. The information from length mutational events could be used in addition to the information from DNA substitutions occurring in the plastid genome. Such changes have been shown to be informative for example in Araliaceae [23], Geraniaceae [24], Poaceae [25] and in early embryopythe lineages [26]. It has been shown that independent gene and intron losses are limited to the more derived monocot and eudicot clades with lineage-specific correlation between rates of nucleotide substitutions, indels, and genomic rearrangements [27].

Here we present the complete chloroplast genome sequence of bittersweet using high-throughput sequencing, as well as the assembly, annotation, gene expression and unique structure characterization of its plastome. We also compare the gene order, inverted repeat (IR) length and examine the variation of structural changes across the family. In order to achieve this we revise the annotations of Solanaceae plastid genome records and correct possible errors. Using this edited plastid genome dataset we present a phylogenetic hypothesis of Solanaceae and examine the distribution of structural changes in the plastid genomes.

## Materials and methods

### Chloroplast isolation

Bittersweet leaves were collected in the Kaisaniemi Botanical Garden of the University of Helsinki, Finland during the summer of 2015. DNA isolation was carried out according to the modified high-salt protocol of Shi et al [28]. DNA concentration was measured with a Qubit fluorometer (Thermo Fisher Scientific, Waltham, MA, USA) and checked on 0.8% agarose gel. We carried out a multiply-primed rolling circle amplification (RCA) according to the protocol of Atherton et al. [29] using a REPLI-g Mini Kit (Qiagen, Hilden, Germany) to produce abundant DNA template.

### Plastid genome sequencing

Paired-end libraries of 300 bp were prepared with Illumina TruSeq DNA Sample prep kit (Illumina, San Diego, CA, USA). Fragment analysis was conducted with an Agilent Technologies 2100 Bioanalyzer using a DNA 1000 chip. Sequencing was carried out on an Illumina MiSeq platform from both ends with 150 bp read length.

### Genome assembly and annotation

Raw reads were first filtered to obtain high-quality clean data by removing low quality reads with a sliding window quality cutoff of Q20 using Trimmomatic [30]. Plastid reads were filtered by reference mapping to Solanaceae plastid genome sequences using Geneious 9.1.7. [31] with medium-low sensitivity and 1,000 iterations. From the collected reads a *de novo* assembly was carried out with the built-in Geneious assembler platform with zero mismatches and gaps allowed among the reads. The similar procedure was conducted with Velvet v1.2.10 [32] with k-mer length 37, minimum contig length 74 and default settings by applying a 400× upper coverage limit. The resulting contigs were then circularized by matching end points. The results of the reference mapping and two de novo methods were compared and inspected. Sanger-based gap closure and IR junction verification was carried out following Moore et al. [33]. Gene annotation was made with a two-step procedure. First we used gene prediction tools DOGMA [34], tRNAscan-SE [35], cpGAVAS [36], Verdant [37] and GeSeq [38] to obtain annotations based on different approaches. In a second step we inspected and curated all annotation manually with comparisons to all published (as of 18.10.2016) plastid genomes of Solanaceae using Geneious. Local BLAST searches were further carried out to confirm the position of CDS regions and genes. We confirmed start and stop codons manually and by comparison to RNA-seq data. For each gene we inspected gene length based on amino acid translations and reconfirmed any internal stop codons. The resulting genome map was drawn with OGDraw v.1.2 [39]. The annotated bittersweet plastid genome was further used as a reference to revise all Solanaceae plastid genomes (deposited by 16.8.2016). Reannotation followed the two-step protocol described above. Plastid genome sequences were transformed into fasta file format then annotated with the software tools [34–38]. All annotations were transferred to Geneious as a new track under the corresponding genome. Sequences were aligned, compared and manually curated compared to bittersweet.

### Genome analyses

Codon frequency and relative synonymous codon usage (RSCU) was calculated on the basis of protein-coding genes using an *in-house* script. We also computed the overall mean of pairwise distances of 80 protein-coding genes of the 32 Solanaceae species based on the Kimura 2-parameter model using MEGA 7.0.21 [40]. Standard error estimate(s) were obtained using bootstrap (1,000 replicates). Complete plastid genome sequences were compared and aligned using mVISTA online tools [41], while the expansion and contraction of the inverted repeat (IR) regions at junction sites was examined and plotted using IRscope [42]. We identified and located repeat sequences (n ≥30 bp and a sequence identity ≥ 90%) found in the bittersweet plastome using REPuter [43]. Repeats larger than 10 bp were classified into the following groups: (i) forward or direct repeats (F), (ii) repeats found in reverse orientation (R), (iii) palindromic repeats forming hairpin loops in their structure (P) and (iv) repeats found in reverse complement orientation (C). Because REPuter overestimates the number of repeats we manually inspected the output file and located the repeats in Geneious. Redundant repeats found entirely within other repeats as well as duplicated parts of tRNAs were pruned. Perfect and compound simple sequence repeats (SSRs) interrupted by 100-bp were located with MISA [44]. A threshold level of seven was applied to mononucleotide repeats, four to dinucleotide repeats and three to tri-, tetra, penta-, and hexanucleotide repeats. Output files were manually edited and exported to Geneious for further inspection.

### Transcriptome analysis and RNA editing site prediction

RNA-seq library files were downloaded from NCBI Short Read Archive for *Solanum dulcamara* (SRR2056039). Reads were mapped to the complete plastid genome and filtered reads were collected with Bowtie 2.0 [45] (mismatch ≤ 2). RNA-seq reads were re-mapped with Geneious using the genome annotation to calculate reads per kilobase per million (RPKM), fragments per kilobase of exon per million fragments mapped (FPKM) and transcripts per million (TPM) for transcript variants. Ambiguously mapped reads were counted as partial matches for each CDS. Putative RNA Editing sites were predicted with an *in silico* approach using the PREP database [46]. Verification of the predicted editing sites was carried out by FreeBayes [47] variant calling.

### Phylogenomic analyses

Our aim was to compare the 32 chloroplast genomes of Solanaceae (data present in NCBI on 16.8.2016) with each other and try to hypothesize when changes have taken place between/among the species and major clades. As outgroup terminals we used *Coffea arabica* L. of Rubiaceae, *Ipomoea batatas* (L.) Lam. and *I*. *purpurea* (L.) Roth. We aligned the 35 complete chloroplast genomes (S1 Table) with MAFFT [48] (S1 Data) since they were lacking inversions or other major changes. We conducted maximum likelihood (ML) analyses using RAxML-NG [49] under three different strategies. 1) One of the IR regions was removed from all plastid genomes to reduce overrepresentation of duplicated sequences then we run RAxML-NG on the unpartitioned alignment under GTR+I+G substitution model as a single partition; 2) The same data matrix was partitioned by gene, exon, intron and intergenic spacer regions (n = 258) and allowed separate base frequencies, α-shape parameters, and evolutionary rates to be estimated for each; 3) we inferred the best-fitting partitioning strategy with PartitionFinder2 [50] for the alignment (n = 24). The best fitting nucleotide substitution models were inferred with jModelTest2 [51]. Branch support values were obtained from 10,000 non-parametric bootstrapping. For each alignment we conducted ten separate runs with RAxML-NG v0.5.0b since log-likelihoods could show variation among individual runs [52]. The complete plastid genome alignment was analyzed also with parsimony as an optimality criterion using the program TNT [53]. The matrix included 19,956 parsimony informative characters and due to its small size we were able to perform analyses using “traditional” search starting from Wagner trees improved using tree bisection reconnection (TBR) algorithm. This search was performed twice with 3,000 replications. We also examined the phylogenetic distribution of structural changes using the tree constructed with parsimony and ML methods implemented in the ancestral state reconstruction tools of Mesquite 3.2 [54]. Major genomic changes were binary coded (S2 Data) and mapped on phylogenetic trees. Phylogenetic trees were visualized and edited with TreeGraph2 [55].

## Results and discussion

### Chloroplast genome assembly and validation

Enriched chloroplast DNA was used to generate 1,645,956 paired-end reads, with an average fragment length of 277 bp, which generated average 1,340 × genome coverage. Low quality reads (Q20) were filtered out, and the remaining high quality reads were utilized in further assembly. For genome assembly we used one reference mapping and two *de novo* methods. As a first step quality filtered reads were mapped to Solanaceae reference genomes, which resulted in an entire contig showing good agreement with published genome sequences. Based on these collected reads we used Geneious and Velvet to produce a single contiguous fragment representing the plastid genome. The three assemblies were compared and discrepancies were manually resolved. With Velvet we obtained a linear contig 43 bp longer (155,623 bp) than with Geneious (155,580 bp) which was caused by a repeated sequence at the start and end point and these were removed. Most *de novo* methods do not account for the circularity of the plastid genome, while Geneious overcomes this by allowing contig circularization during the assembly. The assembly was validated by PCR amplification and Sanger sequencing targeting the four junctions between the IRs and LSC/SSC regions. Sanger results showed identical sequences when compared to the plastid genome demonstrating the accuracy of the assembly. The final chloroplast genome sequence was then submitted to GenBank (KY863443).

### Genome organization, repeats and sequence diversity

**Fig 2:**
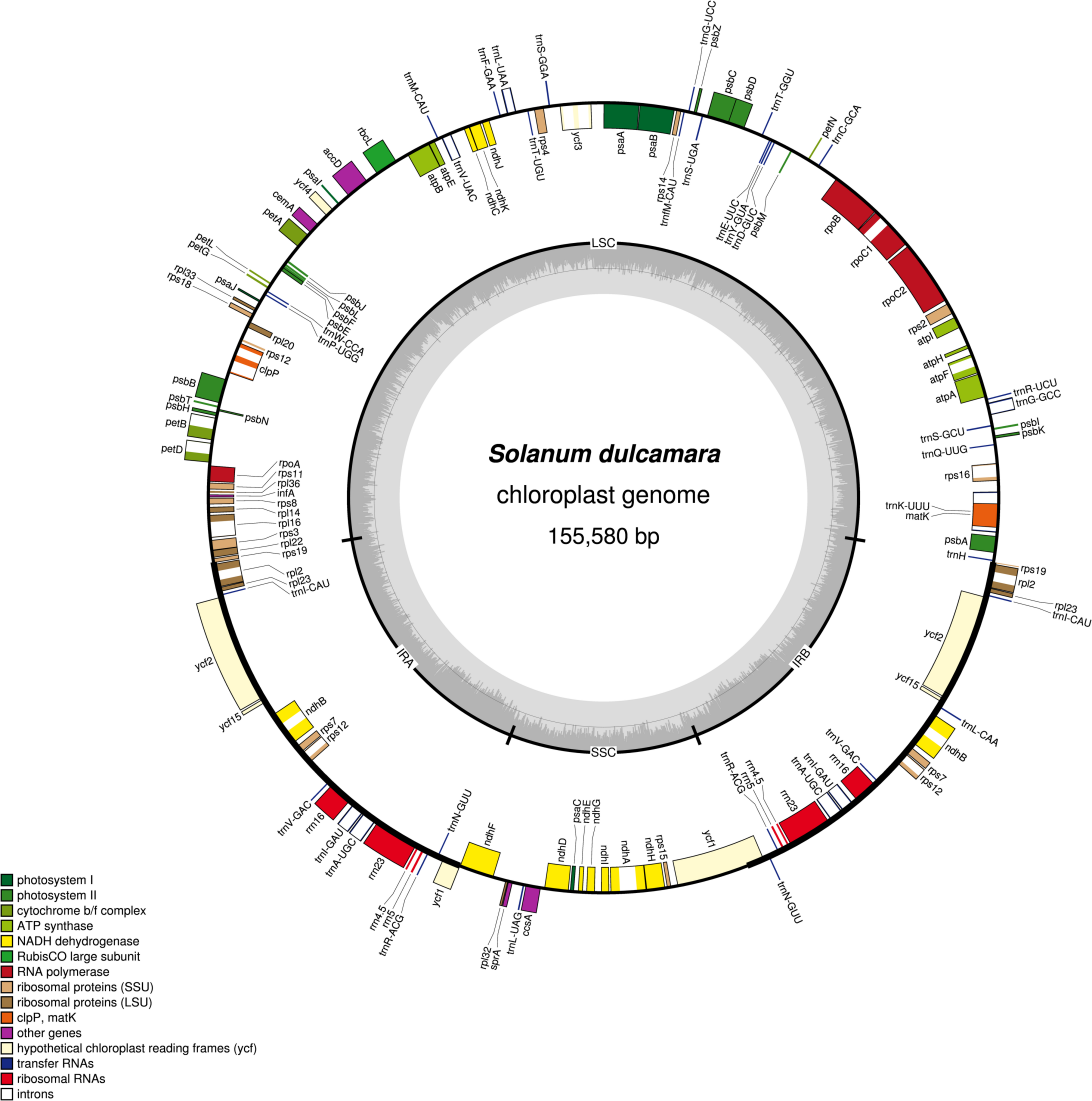
Map of the chloroplast genome of the *Solanum dulcamara*. Genes lying inside of the outer circle are transcribed counterclockwise while those outside that circle are transcribed clockwise. Genes belonging to different functional groups are color coded differently and the GC, AT content of the genome are plotted on the inner circle as dark and light gray, respectively. The inverted repeats, large single copy, and small single copy regions are denoted by IR, LSC, and SSC, respectively.

The chloroplast genome of *Solanum dulcamara* is 155,580 bp long showing a quadripartite structure of long and small single-copy regions of length 85,901 and 18,449 bp, separated with two inverted repeat regions of 25,615 bp (Fig 2). The genome contains 81 protein-coding, 27 tRNA and four rRNA genes comprising the total of 114 unique genes (S2 Table). Seventeen genes contained introns, with *ycf3* and *clpP* containing two. All of these belong to group II introns except *trnL-UAA* with group I intron (S3 Table). The distribution of the genes on different regions of the genome exhibit similarity with other Solanaceae with 13 genes in the SSC and 19 genes in the IR while the rest were on the LSC. The overall GC content of the chloroplast genome is 37.8% resembling other species of Solanaceae (S4 Table). Eighty percent of the total length of the genome is related to genetic regions. The *Arg* amino acid coded with *AGA* codon was the most frequent codon showing RSCU rate of 1,187 (S5 Table).

The majority of the genes show relatively slow evolutionary divergence since all genes had an average sequence distance of less than 0.10 (S6 Table). Low levels of sequence distances indicate the conserved nature of protein-coding genes in Solanaceae. The only gene showing slightly larger distance with a unique function was *spr*A (d = 0.114; S.E = 0.016). Chloroplast genes are mostly subjected to purifying selection and low sequence diversity is due to conservation of the functions of the photosynthetic system. In this context the plastid genome diversity of Solanaceae do not resemble other economically important plant families such as Poaceae where plastid genomes harbor many divergent genes and unique plastid rearrangements [25].

Using MISA we identified 374 SSRs in the bittersweet plastid genome, of which 253 were mono-, 40 di-, 70 tri-, 10 tetra- and one was a pentanucleotide (S7 Table and S3 Data). SSRs were more abundant in the LSC and SSC regions compared to the IRs and 107 occurred in compound formation that were composed of several combinations of SSRs interrupted by maximum distances of 100 bp. The most abundant motifs of the SSRs were poly-A/T stretches characteristic of angiosperm plastid genomes. We also identified 25 larger repeats (> 10 bp) in the bittersweet plastid genome composed of 12 forward, five reverse, five palindromic and three mixed (forward/palindromic) repeats (Table 1) using REPuter. The largest repeat with a size of 83 bp was a forward repeat found in the IGS region of *ycf*3 and *trn*S-GGA. Forward repeats were commonly distributed in the intergenic spacer regions of the genome located mostly in the LSC. Two repeats were found among the introns of *ndh*A, *ycf*3 and *pet*D while one repeat appeared in the *inf*A pseudogene. Three repeats were found among the CDS of *atp*I, *ndh*C and *ycf*2, while another motif was repeated in the *psa*A and *psb*B gene. The repeats in *atp*I and *ycf*2 seem to be conserved since they have also been reported from grasses [25]. The most variable region was the *trn*E-UUC—*trn*T-GGU IGS, which had two palindromic and one forward repeat.

**Table 1:** Repeat sequences of the *Solanum dulcamara* chloroplast genome.

No	Type	Location		Region		Repeat unit	Period size (bp)	Copy Nr.
1	F	*ycf*3—*trn*S-GGA	IGS	LSC	AACAATTTTAAAGAAAAATTGTATCTTTATCCCGGAGTC TTGAAGGAAAGAAAAATGGTTCTTTGTTTTGACTTTGATGAAA		83	2
2	F	*psa*A and *psb*B	CDS	LSC	TGCAATAGCTAAATGGTGATGGGCAATATCAGTCAGCC		38	2
3	F	*ndh*A and *ycf*3	intron	LSC/SSC	CAGAACCGTACGTGAGATTTTCACCTCATACGGCTCCT		38	2
4	F	*inf*A	pseudogene	LSC	AGGTATCAACTAATCTAATCCAATTTGGATATTATAAA		38	2
5	F	*atp*B—*rbc*L	IGS	LSC	TTAGCACTCGATGAGACTGAGTTAATTTGCAAGCT		34	2
6	F	*psb*A—*ycf*3—*trn*S-GGA	IGS	LSC	TTAATATAATAAAAAGAAGTCTATTTTGT		29	2
7	F	sprA—*trn*L-UAG	IGS	SSC	CCTTTTTAACTCTATTCCTTAATTGAGT		28	2
8	P	*rps*12—*trn*V-GAC	IGS	IR	TGAGATTTTCACCTCATACGGCTCCT		26	2
9	P	*pet*D	intron	LSC	TATAAGTGAACTAGATAAAACGGAAT		26	2
10	F	*trn*G-GCC—*trn*R-UCU	IGS	LSC	TTAGTACATCATTGAATATACAA		23	2
11	F	*psa*J—*rpl*33	IGS	LSC	GTGGACGGGCTGAGGAATGGGG		22	2
12	F/P	*rps*12—*trn*V-GAC	IGS	IR	ATTAGATTAGTATTAGTTAGT		21	4
13	F	*ndh*C—*trn*V-UAC	IGS	LSC	TCCTTTTATTATTATTTAAT		20	2
14	P	*psb*T—*psb*N	IGS	LSC	AGTTGAAGTACGGAGCCTCC		20	2
15	F	*trn*E-UUC—*trn*T-GGU and *rps*4—*trn*T-UGU	IGS	LSC	TTATTTAGTATTTCGAATT		19	2
16	F/P	*ycf*2	CDS	IR	CGATATTGATGATAGTGAC		19	4
17	F	*rps*16—*trn*Q-UUG	IGS	LSC	ATTATAATATTAATTA		16	3
18	P	*trn*E-UUC—*trn*T-GGU	IGS	LSC	TTTTATTTAGAAA		13	2
19	P	*trn*E-UUC—*trn*T-GGU	IGS	LSC	CATCATACTATGA		13	2
20	R	*trn*F-GAA—*ndh*J	IGS	LSC	TCTCCTCTTTT		11	2
21	R	*ndh*C	CDS	LSC	CATCAAAAACA		11	2
22	R	*atp*H—*atp*I	IGS	LSC	TTTATTATTTA		11	2
23	R	*atp*I	CDS	LSC	ACAAAAATAA		11	2
24	R	*pet*L—*pet*G	IGS	LSC	CCTCTTTTTT		10	2
25	F/P	*rps*12—*trn*V-GAC	IGS	IR	AACTAATACT		10	6

### Reannotation of Solanaceae plastid genomes

We noticed a litany of errors in currently deposited annotations, which were corrected for our analyses in a two-step curation process using gene prediction tools followed by manual adjustments. The reannotated genome files could be accessed as an online supplement (S4 and S5 Data). We provide here the first annotation for the sequences of *S*. *pennellii* Correll and *Iochroma loxense* (Kunth) Miers, which entirely lacked genome features. A complete list of annotation errors is found in S8 Table, and illustrates the difficulties encountered when attempting to compare across genomes. These differences could cause considerable consequences inferring gene functionality or synteny. In general annotations of the LSC and SSC corresponding to the basic quadripartite structure of angiosperm plastid genomes were entirely missing or sparsely indicated. Inverted repeats (IRs) were either unannotated or their orientation, size and correct naming was erroneous. Compared to the tobacco reference order LSC-IRB-SSC-IRA [56], the erroneous annotation LSC-IRA-SSC-IRB is often applied. It is important to note that the IR sequences of the *Atropa belladonna* L. and *Saracha punctate* Ruiz. c Pav. were dissimilar. Inverted repeat sequences are under concerted evolution [22] and divergent sequences could be possible sequencing/assembly errors in these two genomes or they could represent a relatively rare case of chloroplast evolution. Several protein-coding genes had errors with assigned start/stop codons. For example, the start codon of the *rpo*C2 gene is shifted with 12 bps in most deposited plastid genomes except in *Nicotiana* L. species and in *Datura stramonium* L. Annotations were found to be insufficient for genes containing introns since they were lacking exon and/or intron designations. The exon-intron boundaries had variable annotation for many genes with high level of synteny, e.g., *atp*F or *rpo*C1. Gene annotations were missing for some species in case of *psb*K and *psb*Z, while the later was often annotated as *ihb*A now regarded as a synonym of *psb*Z.

Besides previously described genes we located and annotated hypothetical gene *ycf*68 the 218 bp long small plastid RNA (*spr*A) gene in all studied genomes. Homologs of *spr*A are present in eudicots but absent from monocots and they are rarely annotated in plastid genomes. This gene was reported to play a role in the 16S rRNA maturation in *Nicotiana tabacum* L. [57], but its function is non-essential under normal growth conditions [58]. It is not part of the catalytic core nor does it guide the rRNA machinery rather it acts independently. In this respect its function is similar to other non-essential plastid spRNAs.

According to our experiences during the reannotation none of the currently existing tools provided submission ready annotations. They required minor or even extensive manual curation especially with the most commonly used DOGMA producing results which require expert interpretation and laborious adjustments. For example annotating intron-containing genes or genes with short exons such as *pet*B, and dealing with trans-splicing reading frames like *rps*12 is challenging with DOGMA. Moreover DOGMA [34] generates a special output file compared to CpGAVAS [36] or GeSeq [38], which generate standard general feature format (.gff) or GenBank (.gb) files that can be integrated with other software without further processing. From the currently available tools GeSeq [38] generated the highest quality results by annotating >95% of the genes and coding regions correctly compared to our curated reference set. In most cases annotation errors were propagated from erroneous references to newly assembled genomes creating a systematic problem in Solanaceae. For future reference we advise the jettison of outdated annotation tools such as DOGMA and advise the use of up-to-date novel software such as GeSeq to avoid complications. For de novo sequenced Solanaceae plastid genomes bittersweet can also serve as a novel reference for comparison and annotation.

### Expansion and contraction of IR regions

**Fig 3:**
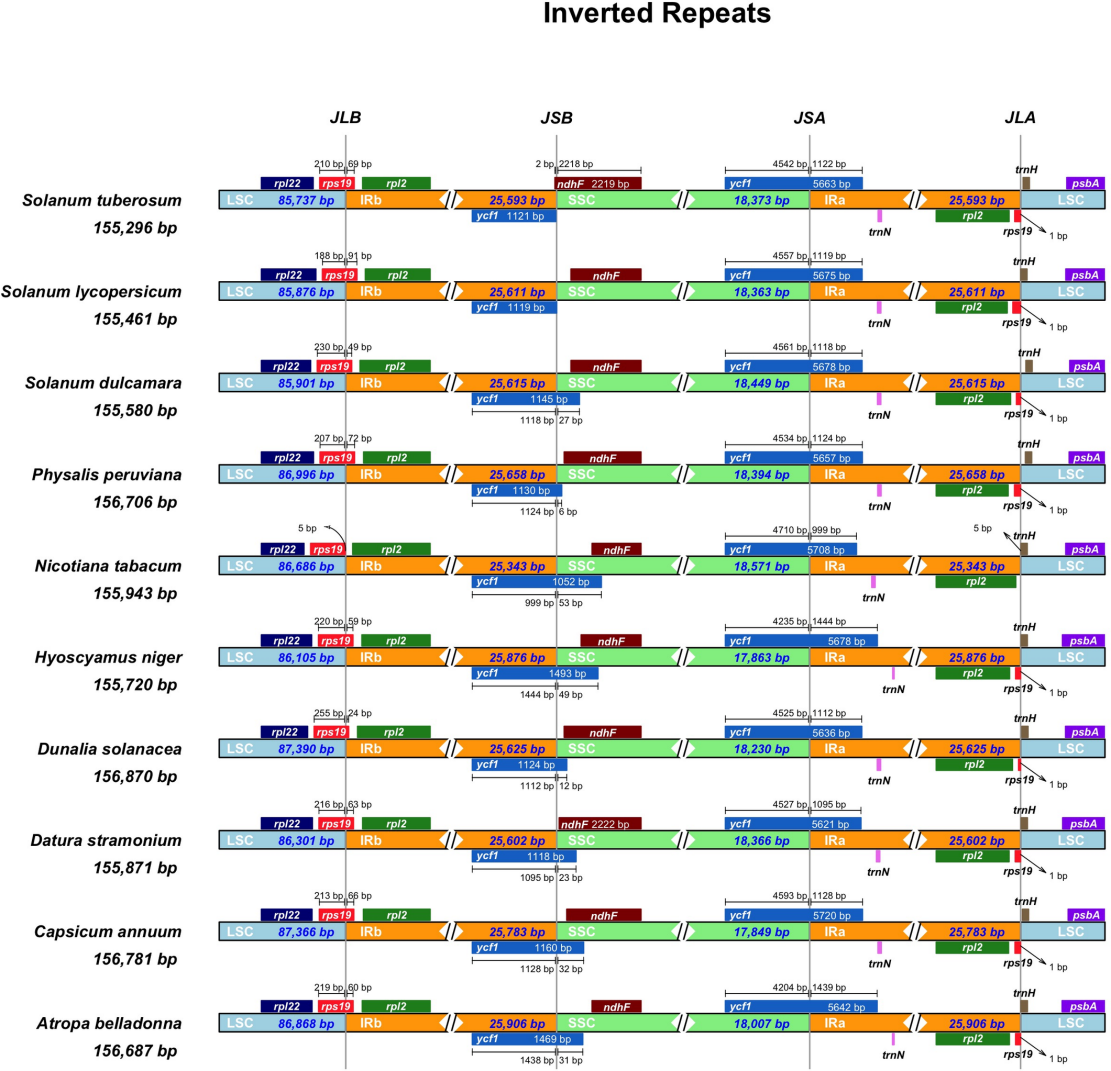
Junction sites of the inverted repeats. For each species, genes transcribed in positive strand are depicted on the top of their corresponding track with right to left direction, while the genes on the negative strand are depicted below from left to right. The arrows are showing the distance of the start or end coordinate of a given gene from the corresponding junction site. For the genes extending from a region to another, the T bar above or below them show the extent of their parts with their corresponding values in base pair while nothing is plotted for the genes tangent to the sites. The plotted genes and distances in the vicinity of the junction sites are the scaled projection of the genome. JLB (IRb /LSC), JSB (IRb/SSC), JSA (SSC/IRa) and JLA (IRa/LSC) denote the junction sites between each corresponding two regions on the genome.

By using the curated genome annotations we compared the junction sites of ten selected Solanaceae plastid genomes. In general IRs are systematically un-annotated in deposited plastid genomes with several genes, for example *rp*l2, missing. Pseudogenes like the truncated ψ*rps*19 are mislabeled or entirely missing, which made the comparison of the IR regions cumbersome and time consuming. Therefore, we utilized an *in house* script, IRscope [42] to overcome these problems, and located the IRs and plotted the genes in vicinity of the junctions (Fig 3). The length of the IR regions were similar ranging from 25,343 bp to 25,906 bp showing some expansion. The endpoint of the Solanaceae JLA is characteristically located upstream of the *rps*19 and downstream of the *trn*H-GUG. In Solanoideae, the IR expanded to partially include *rps*19 creating a truncated ψ*rps*19 copy at JLA, thus this pseudogene is missing from *Nicotiana*. The extent of the IR expansion to *rps*19 varies from 24 to 91 bp and the end point seems to be conserved not exceeding to the following intergenic spacer region. Furthermore, *infA*, *ycf15*, and a copy of *ycf1* located on the JSB were detected as pseudogenes. In contrast to *Solanum tuberosum* and *S*. *lycopersicum* where JSB is tangent to the end of the pseudo *ycf1* gene, the copy of this gene in *S*. *dulcamara* is showing an extra part extended further to the SSC (Fig 3).

### Phylogenetic relationships in Solanaceae

**Fig 4:**
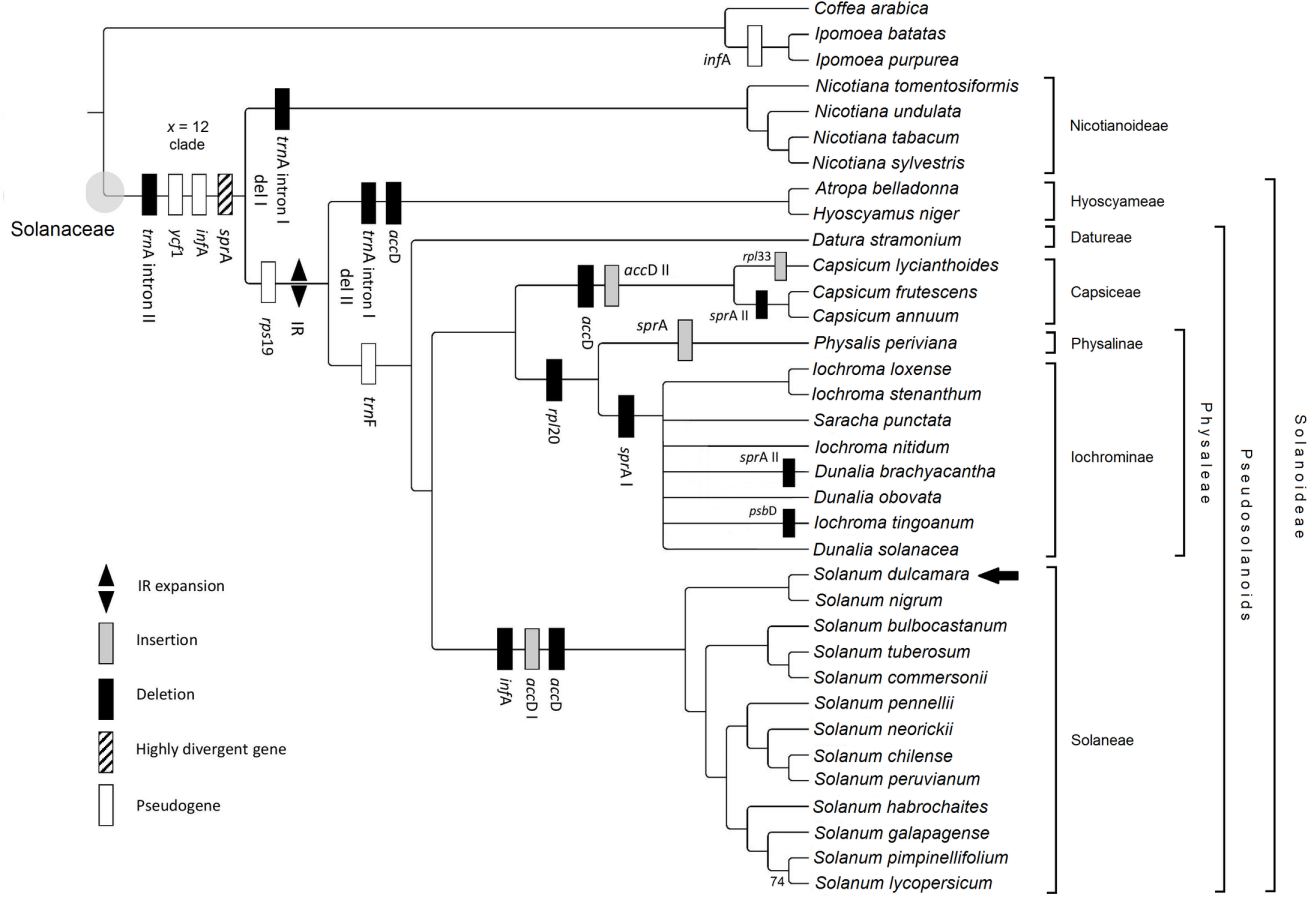
Cladogram illustrating the phylogenetic relationships of Solanaceae based on complete chloroplast genome sequences. Plastid genome rearrangement events are mapped on the branches of the best scoring maximum likelihood tree generated with RAxML-NG. Each node has 100% bootstrap support value. A node with lower support value indicated and those with support values below 50% collapsed. Currently recognized suprageneric groups are listed on the right.

Our phylogenetic analyses of the whole plastid genome alignment resulted in highly resolved trees (Fig 4), with almost all clades recovered having maximum branch support values (S1 Fig). We conducted phylogenetic analysis with three different partitioning strategies under maximum likelihood and analyzed the matrix also using parsimony. All our analyses resolved similar topologies which confirm results of previous phylogenetic analyses based on fewer genes [10, 59] but in several cases groups with low support values of earlier studies are resolved in our tree with high support values.

Trees of parsimony and ML analyses are congruent except for the clade composed of iochromas (S1 Fig). Iochrominae is a diverse clade of Physaleae with ca. 34 species and six traditionally recognized genera, including *Acnistus* Schott, *Dunalia* Kunth, *Eriolarynx* (Hunz.) Hunz, *Iochroma* Benth., *Saracha* Ruiz & Pav. and *Vassobia* Rusby. Members of this group are shrubs of high elevation in the Andes displaying great diversity in floral characteristics and pollination system. Recent molecular phylogenetic studies resolved Iochrominae with high support value but relationships within the clade have remained poorly resolved [10, 59]. In this group nodal resolution does not scale proportionately to the length of sequence analyzed, and structural variations in the plastid genome seem to be accumulated as compared to other clades.

Iochrominae represented here by *Iochroma*, *Dunalia* and *Saracha* appear to be monophyletic based on the analyses of the complete chloroplast genome sequences. However, our results also suggest that two of these morphologically delimited genera (*Iochroma* and *Dunalia*) are not monophyletic. Smith and Baum [60] utilizing nuclear markers (ITS, *waxy* and LEAFY) also found that generic boundaries are not congruent with the current taxonomy. Iochromas might have highly reticulated history that is impossible to be represented by a dichotomic tree. The unequivocal resolution of iochromas will likely require the inclusion of nuclear genomic regions.

We resolved *Solanum dulcamara* in a separate clade with *S*. *nigrum* appearing as a sister group. This reinforces the close relationship of the Dulcamaroid and Morelloid clades as proposed by other molecular phylogenetic analyses based on fewer markers [8–10]. The informally named *x* = 12 clade is found in our analysis as sister to Nicotianoideae. In this group the chromosome numbers are based on 12 pairs [61], and members are estimated to have gone through two separate whole-genome duplication (WGD) events ca. 117 Ma [62] and 49 Ma BP [63], respectively. Increased sampling outside this group is needed since this could shed light on ancient WGDs in the family. Plastid genomes of Solanaceae hold much promise for resolving relationships among clades of the family that have previously been problematic. Although the phylogenomic tree presented in this study is largely robust it should be kept on mind that our sampling is still sparse in terms of the number of terminals. It is also important to note that organellar phylogenomics may fail in rapidly radiating groups with interspecific hybridization as exemplified here by iochromas. Other biological processes such as incomplete lineage sorting might also make phylogenetic analyses very difficult, however, organellar phylogenomics can be used to detect such processes.

### Plastid genome structure of Solanaceae

Intending to identify and map the major structural changes of Solanaceae plastid genomes on the phylogenetic tree, we selected ten Solanaceae plastid genomes for detailed comparison representing diverse groups of the family and included two outgroup taxa in the analysis. Gene comparisons were extended to the entire Solanaceae dataset using local alignments with MAFFT and the curated genome annotations. The size of the plastid genomes varied between 155,312 bp (*Solanum tuberosum*) to 162,046 bp (*Ipomoea purpurea*) (S4 Table). Our comparison shows that gene content and synteny are highly conserved across Solanaceae plastid genomes (S2 Fig). All species analyzed display complete gene synteny when accounting for expansion and contraction of the IRs (Fig 3). The organization and evolution of Solanaceae plastid DNA have been analyzed by previous studies using restriction site methods [64], PCR surveys [65–68] and complete genome sequences [69–74]. These comparisons highlighted some features of Solanaceae but the phylogenetic distribution of these rearrangements have not been examined. Our comprehensive comparison of complete chloroplast genomes of ten Solanaceae and *S*. *dulcamara* confirm the presence of all the genomic rearrangements reported previously. We will briefly review the conclusions made before and then highlight the novel aspects resulting from our analysis and moreover, examine the distribution of these structural changes using the phylogenetic hypothesis constructed based on complete plastid genome alignment.

We observed ten characteristic features in Solanaceae plastid genomes linked to indels or pseudogenization processes (Table 2). Two genes, one copy of ψ*ycf*1 and ψ*rps*19 at the IRb/SSC and IRa/LSC junction were truncated pseudogenes, while *inf*A has become non-functional through partial degradation. The substitutions of *inf*A orthologues in Solanaceae show almost equal numbers of substitutions at all codon positions with missing start codons. It is also a pseudogene in *Ipomoea* representing Convolvulaceae, the sister family of Solanaceae but it appears to be functional in *Coffea* of Rubiaceae [75] used as a distant outgroup of Lamiids. The *inf*A gene seem to have become non-functional in the ancestor of Solanales multiple times independently. In Solanaceae the pseudogenization further continued with a monophyletic 124-bp deletion in the ancestor of the genus *Solanum*. Further changes appeared in four protein-coding genes; there is a 64-bp deletion in *psb*D of *Iochroma tingoanum* while 31-bp was deleted from the *rpl*20 gene in members of Physaleae. *Capsicum lycianthoides* Bitter had a unique 15-bp insertion in the *rpl*33 gene. The *acc*D gene, which encodes one of the four subunits of the acetyl-CoA carboxylase enzyme in most chloroplasts show a 24-bp insertion in the members of the ‘x = 12 clade’ [61]. This seems to be an ancestral trait shared by members of Nicotianoideae and Solanoideae and maintained in *Datura* L., *Nicotiana*, *Physalis* L. and Iochromas but lost independently in *Hyoscyamus* L., *Capsicum* L. and *Solanum*. The latter two went through a characteristic 141-bp and a small 9-bp insertion. The 141-bp deletion was also confirmed in *Capsicum* by Jo et al. [72]. The small plastid RNA (*spr*A) gene, which includes a complementary segment to the pre-16S rRNA shows high variability among Solanaceae. Functional *spr*A copies were present in most Solanaceae but several mutation event indicate it has be non-functional is some groups. A 52-bp deletion appeared in *Capsicum* at the 5’ and further 37-bp were deleted in iochromas while *Physalis* showed an autapomorphic 14-bp insertion (S3 Fig). The function *spr*A has been lost independently multiple times once in Iochrominae and in Capsaceae, however, the gene remained functional in *Capsicum lycianthoides*.

**Table 2:** Major changes in the chloroplast genomes of Solanaceae.

Gene	Insertion	Deletion	Pseudogene	Notes
*acc*D	2	1	-	24-bp deletion in the 'x = 12 clade' except (*Nicotiana*, *Datura*, *Physalis*, Iochromas) 141-bp insertion in *Capsicum* 9-bp insertion in *Solanum*
*inf*A	-	1	+	124-bp deletion in *Solanum*
*psb*D	-	1	-	64-bp deletion in *Iochroma tongoanum*
*rpl*20	-	1	-	31-bp deletion Physaleae
*rpl*33	1		-	15-bp insertion in *Capsicum lycianthoides*
*rps*19	-	-	+	-
*spr*A	1	2	-	14-bp insertion in *Physalis* 52-bp deletion in *Capsicum* 37-bp deletion Iochromas
*trn*A-UGC	-	2	-	108-bp and 141-bp intron deletion in *Nicotiana* and Atropa/Hyosciamus
*trn*F-GAA	-	-	+	Uniting a group of Pseudosolanoids
*ycf*1	-	-	+	Truncated pseudogenization of one ycf1 copy in Solanaceae.

Genomic changes also affect tRNA genes and neighboring regions. The most notable change is the duplication of the original phenylalanine (*trn*F-GAA) gene in a tandem array composed by multiple pseudogene copies in Solanaceae. The pseudogene copies are composed of several highly structured motifs that are partial residues or entire parts of the anticodon, T- and D-domains of the original *trn*F gene [66]. Previously it was shown that these copies are subjected to possible inter- or intrachromosomal recombination events [67] and they have high taxonomic relevance uniting a unique plastid clade of Pseudosolanoids [68]. They provide support for previous results [10, 59] separating the Atropina and Juanulloae clades from Solaneae, Capsaceae, Physaleae, Datureae and Salpichroina [68]. Another tRNA related structural change is apparent in the group II intron of *trn*A-UGC, where 108-bp was deleted in *Nicotiana* and extended up to 147-bp in *Atropa* L. and *Hyoscyamus*.

### Gene expression analyses

We carried out the expression analysis of 85 protein-coding genes (Table 3). As we were mostly interested about CDS/gene features we used only these annotation types for read mapping. We also used the RNA-seq data set to verify start/stop codon positions and further ultimate or penultimate editing sites from the reannotation process. A total of 147,721 reads were mapped to the bittersweet plastid genome with an average 112× read depth. The largest portion of reads 25,910 (17.53%) and 12,582 (8.51%) was derived from adenosine triphosphate (ATP) synthase genes and from the photosystem II (PSII) complex. All genes were normally expressed while the five most abundant were *atp*B, *atp*E, *clp*P, *rps*7 and *psb*M (>10,000 FPKM). The assembled consensus sequence from the mapped reads (148,110 bp long) covered 95.22% of the genome spanning through also intergenic spacer (IGS) sequences. Accordingly, a nearly complete pseudo *Solanum dulcamara* plastid genome was unexpectedly obtained by means of transcriptome data. We found multiple transcripts mapping to several non-functional genes for example *ycf*15, *inf*A, or to truncated pseudogenes ψ*ycf*1 and ψ*rps*19 at the JLA (IRa/LSC). From these *inf*A, ψ*ycf*1 and ψ*rps*19 were nearly completely covered (S4 Fig) showing that they are indeed transcribed, while *ycf*15 had sparse coverage. This indicates that transcriptome sequencing captured both primary and processed mRNA sequences of the plastome. The detected and mapped reads of the bittersweet plastid RNA population could be grouped into three major types i) mRNAs ii) non-coding RNAs from IGS regions and iii) tranditonal non-coding RNAs (rRNAs and tRNAs). Similar patterns were observed by Shi et al. [76] and also in earlier studies using Northern blot hybridization where 90% of the plastid genome was found to be transcribed [77]. Such patterns could be caused by transcriptional uncoupling of genes in polycistronic clusters [78]. Non-coding RNAs (ncRNAs) in the plastome are further transcribed from intergenic regions (IGSs), which play important role in post-transcriptional regulation [79]. Cyanobacteria contain several ncRNAs making it plausible that also plastomes harbor a wide variety of undetected regulatory ncRNAs [80]. These results show that non-functional genes are transcribed as a precursor polycistronic transcript, which are later edited during pre-mRNA maturation. In order to activate the function of other genes plastid primary transcripts are edited and expression in the plastome mainly occurs at a post-transcriptional stage. The multiple transcription arrangement leading to the full transcription of plastid genomes is a prokaryotic ancestral trait still preserved in eukaryotic cells billion years after the primary endosymbiosis [81, 82].

**Table 3:** RNA Expression of protein-coding genes in the *Solanum dulcamara* chloroplast genome. Reads per kilobase per million (RPKM), fragments per kilobase of exon per million fragments mapped (FPKM) and transcripts per million (TPM) for transcript variants.

Gene	Location min.	Max	Length	FPKM	RPKM	TPM
*atp*B	54,285	55,781	1,497	278926.7	278926.7	232422.2
*atp*E	53,887	54,288	402	238932.2	238932.2	199095.9
*clp*P	71,842	73,864	591	120109.6	120109.6	100084.1
*rps*7	142,238	142,705	468	91956.3	91956.3	76624.7
*rps*7	98,701	99,168	468	88572.2	88572.2	73804.8
*psb*M	30,605	30,709	105	22431.7	22431.7	18691.7
*psb*A	552	1,613	1,062	21738.5	21738.5	18114.1
*ycf*1	125,388	131,069	5,682	21573.2	21573.2	17976.3
*psb*K	7,750	7,935	186	21287.0	21287.0	17737.9
*psa*J	68,897	69,031	135	19101.3	19101.3	15916.6
*rbc*L	56,597	58,030	1,434	13932.8	13932.8	11609.9
*rpl*20	70,391	70,777	387	11700.0	11700.0	9749.3
*psb*I	8,248	8,406	159	11493.2	11493.2	9576.9
*rps*12	71,590	142,184	372	11407.7	11407.7	9505.7
*rps*12	71,590	100,015	372	11243.9	11243.9	9369.3
*psb*J	65,856	65,978	123	10565.0	10565.0	8803.5
*atp*F	11,989	13,234	555	8579.1	8579.1	7148.8
*psb*E	66,378	66,629	252	8540.8	8540.8	7116.8
*rps*16	5,077	6,199	267	7528.7	7528.7	6273.4
*atp*H	13,637	13,882	246	7180.9	7180.9	5983.6
*ycf*1	110,382	111,527	1,146	6963.1	6963.1	5802.2
*rps*18	69,855	70,160	306	6768.2	6768.2	5639.8
*rps*15	124,723	124,986	264	6537.5	6537.5	5447.5
*rps*19	85,655	85,933	279	6258.8	6258.8	5215.3
*rpl*22	85,135	85,602	468	6225.9	6225.9	5187.8
*rps*14	38,024	38,326	303	6098.1	6098.1	5081.4
*ndh*H	123,425	124,606	1,182	5531.4	5531.4	4609.1
*psb*T	76,034	76,138	105	5511.2	5511.2	4592.4
*rpl*16	82,913	84,349	405	5113.7	5113.7	4261.1
*psb*Z	37,053	37,241	189	4941.9	4941.9	4117.9
*psa*C	118,619	118,864	246	4704.7	4704.7	3920.3
*cem*A	62,915	63,604	690	4472.9	4472.9	3727.1
*rps*3	84,494	85,150	657	4249.4	4249.4	3540.9
*ycf*3	43,702	45,689	507	4245.1	4245.1	3537.4
*psb*C	34,984	36,369	1,386	4211.8	4211.8	3509.6
*psb*B	74,308	75,834	1,527	4135.4	4135.4	3445.9
*rpl*33	69,463	69,663	201	3838.7	3838.7	3198.7
*ndh*A	121,171	123,423	1,092	3830.3	3830.3	3191.7
*rpl*2	153,916	155,406	825	3704.0	3704.0	3086.5
*psa*B	38,445	40,649	2,205	3480.8	3480.8	2900.4
*pet*N	29,403	29,492	90	3384.1	3384.1	2819.9
*psa*A	40,675	42,927	2,253	3343.5	3343.5	2786.1
*rpl*2	86,000	87,490	825	3322.6	3322.6	2768.6
*psb*D	33,939	35,000	1,062	3259.8	3259.8	2716.3
*pet*B	76,806	78,207	652	3238.8	3238.8	2698.8
*ndh*K	50,792	51,535	744	3097.5	3097.5	2581.1
*ndh*I	120,574	121,077	504	2860.4	2860.4	2383.5
*ndh*B	96,202	98,413	1,533	2834.4	2834.4	2361.9
*ndh*B	142,993	145,204	1,533	2794.7	2794.7	2328.7
*rps*2	16,048	16,758	711	2770.1	2770.1	2308.3
*atp*A	10,411	11,934	1,524	2618.0	2618.0	2181.5
*atp*I	15,056	15,799	744	2565.4	2565.4	2137.6
*rps*8	81,838	82,242	405	2506.7	2506.7	2088.8
*rpl*14	82,410	82,778	369	2366.1	2366.1	1971.6
*ndh*J	50,210	50,686	477	2256.1	2256.1	1879.9
*psb*N	76,212	76,343	132	2230.4	2230.4	1858.6
*ndh*C	51,526	51,888	363	2097.6	2097.6	1747.9
*pet*D	78,398	79,611	483	1639.5	1639.5	1366.2
*psb*H	76,455	76,676	222	1554.9	1554.9	1295.6
*ndh*G	119,645	120,175	531	1453.1	1453.1	1210.8
*mat*K	2,136	3,665	1,530	1446.5	1446.5	1205.4
*pet*G	67,909	68,022	114	1424.9	1424.9	1187.3
*rpo*C1	21,302	24,105	2,067	1414.5	1414.5	1178.7
*rps*11	80,882	81,298	417	1412.1	1412.1	1176.6
*pet*A	63,824	64,786	963	1244.0	1244.0	1036.6
*rpo*A	79,803	80,816	1,014	1201.5	1201.5	1001.1
*ycf*4	61,594	62,148	555	1170.7	1170.7	975.5
*ndh*E	119,116	119,421	306	1128.0	1128.0	940.0
*rpl*23	153,616	153,897	282	1116.0	1116.0	930.0
*psa*I	61,037	61,147	111	1097.5	1097.5	914.6
*rpl*23	87,509	87,790	282	1080.0	1080.0	900.0
*rpl*32	114,524	114,691	168	966.9	966.9	805.7
*acc*D	58,765	60,288	1,524	906.0	906.0	754.9
*rps*4	46,706	47,311	606	770.6	770.6	642.1
*rpo*B	24,111	27,338	3,228	673.0	673.0	560.8
*pet*L	67,627	67,722	96	634.5	634.5	528.7
*ndh*D	116,999	118,501	1,503	580.9	580.9	484.1
*rpo*C2	16,983	21,149	4,167	438.5	438.5	365.4
*rpl*36	81,400	81,513	114	356.2	356.2	296.8
*ycf*2	88,118	94,960	6,843	308.6	308.6	257.1
*ycf*2	146,446	153,288	6,843	281.9	281.9	234.9
*ndh*F	111,507	113,729	2,223	246.6	246.6	205.5
*ccs*A	115,826	116,767	942	215.5	215.5	179.6
*ycf*15	95,045	95,308	264	76.9	76.9	64.1
*ycf*15	146,098	146,361	264	76.9	76.9	64.1

### Plastid RNA editing

Chloroplast RNA editing was first discovered in 1991 [83] and it could be defined as the post-transcriptional modification of pre-RNAs by insertion, deletion or substitution of specific nucleotides to form functional RNAs. In the plastid genome this processing machinery is crucial to alter the long pre-RNA transcripts as detailed above. The most frequent editing events in plants are C-to-U changes, however, U-to-C editing has also been observed [84]. RNA editing is absent in liverworts and green algae while it is abundant in lycophytes, ferns and hornworts [85]. To gain insight to the RNA metabolism of bittersweet we first predicted 28 RNA editing sites out of 35 plastid genes with PREP (Table 4). We aligned RNA read sequences using bittersweet as a reference genome and by variant searching we confirmed 23 editing sites from those predicted with PREP. We found four additional editing sites with variant search not detected by PREP resulting in 27 confirmed editing sites. From these 25 (92.5%) were C-to-U changes and two were A-to-G and G-to-U conversions resulting in non-synonymous amino acid changes. The percentage of conversion rates for each edit varied between 25 to 95.9% according to the calculated ratio between the numbers of reads with an alternate base compared with the reference. Some edits showed high rates (>90%) for *atp*F, *ndh*B, *pet*B, *psb*E and *rps*14 genes making it clear that these forms are highly abundant among processed RNAs in bittersweet. Edits of these particular genes has also been reported in previous studies of embryophytes [86, 87] suggesting the conserved feature of such sites. It has been proposed that RNA editing is of monophyletic origin and evolved as a mechanism to conserve certain codons [88]. For example the start codon (AUG) of the *psb*L and *ndh*D is RNA edited (C-to-U) in all Solanaceae except in *Datura stramonium* where the start codon of *psb*L remains unedited.

**Table 4:** RNA editing sites in the *Solanum dulcamara* chloroplast genome.

Gene Name	Length	Strand	Region	Nt pos	AA pos	Effect	Nt Change	Score	RNASeq	PREP	Number of reads
*atp*F	1246	+	LSC	92	31	CCA (P) = > CUA (L)	C = > U	0.86	+	+	U; 49 (90.7%), C; 5 (9.3%)
*ndh*A	2258	+	SSC	341	114	UCA (S) = > UUA (L)	C = > U	1	+	+	U; 35 (70%), C; 15 (30%)
*ndh*A	2258	+	SSC	566	189	UCA (S) = > UUA (L)	C = > U	1	+	+	U; 20 (34.4%), C; 38 (65.6%)
*ndh*A	2258	+	SSC	1073	358	UCC (S) = > UUC (F)	C = > U	1	+	+	U; 49 (74.2%), C; 17 (25.8%)
*ndh*B	2212	+	IR	149	50	UCA (S) = > UUA (L)	C = > U	1	+	+	U; 33 (86.8%), C; 5 (13.1%)
*ndh*B	2212	+	IR	467	156	CCA (P) = > CUA (L)	C = > U	1	+	+	U; 34 (87.1%), C; 5 (12.9%)
*ndh*B	2212	+	IR	586	196	CAU (H) = > UAU (Y)	C = > U	1	+	+	U; 26 (82.3%), C; 8 (17.7%)
*ndh*B	2212	+	IR	611	204	UCA (S) = > UUA (L)	C = > U	0.80	+	+	U; 33 (89.1%), C; 4 (10.9%)
*ndh*B	2212	+	IR	737	246	CCA (P) = > CUA (L)	C = > U	1	+	+	U; 47 (95.9%), C; 2 (4.1%)
*ndh*B	2212	+	IR	746	249	UCU (S) = > UUU (F)	G = > U	1	+	+	U; 40 (95.2%), C; 2 (4.8%)
*ndh*B	2212	+	IR	780	260	UGG (P) = > UGU (C)	C = > U	-	+	-	U; 32 (50.8%), G, 31 (49.2%)
*ndh*B	2212	+	IR	830	277	UCA (S) = > UUA (L)	C = > U	1	+	+	U; 44 (97.1%), C; 1 (2.9%)
*ndh*B	2212	+	IR	836	279	UCA (S) = > UUA (L)	C = > U	1	-	+	-
*ndh*B	2212	+	IR	1481	494	CCA (P) = > CUA (L)	C = > U	1	+	+	U; 20 (52.6%), C; 18 (47.4%)
*ndh*D	1504	+	SSC	2	1	ACG (T) = > AUG (M)	C = > U	-	+	-	U; 40 (95.2%), C; 2 (4.8%)
*ndh*F	2223	+	SSC	290	97	UCA (S) = > UUA (L)	C = > U	1	-	+	-
*pet*B	1398	-	LSC	1168	390	CGG (R) = > UGG (W)	C = > U	1	+	+	U; 15 (93.8%), C; 1 (6.2%)
*pet*B	1398	-	LSC	1361	454	CCA (P) = > CUA (L)	C = > U	1	+	+	U; 23 (74.2%), C; 8 (25.8%)
*psb*E	252	+	LSC	214	72	CCU (P) = > UCU (S)	C = > U	1	+	+	U; 112 (93.3%), C; 8 (6.7%)
*psb*L	124	+	LSC	2	1	ACG (T) = > AUG (M)	C = > U	-	+	-	U; 40 (95.2%), C; 2 (4.8%)
*rpl*20	387	+	LSC	308	103	UCA (S) = > UUA (L)	C = > U	0.86	+	+	U; 107 (56.6%), C; 82 (43.4%)
*rpo*A	1014	+	LSC	830	277	UCA (S) = > UUA (L)	C = > U	1	+	+	U; 8 (61.5%), C; 5 (38.5%)
*rpo*A	1014	+	LSC	903	301	AUG (M) = > GUG (V)	A = > G	-	+	-	G; 25 (62.5%), A; 15 (37.5%)
*rpo*B	3213	+	LSC	338	113	UCU (S) = > UUU (F)	C = > U	1	+	+	U; 15 (75%), C; 5 (25%)
*rpo*B	3213	+	LSC	473	158	UCA (S) = > UUA (L)	C = > U	0.86	+	+	U; 13 (76.5%), C; 4 (23.5%)
*rpo*B	3213	+	LSC	551	184	UCA (S) = > UUA (L)	C = > U	1	-	+	-
*rpo*B	3213	+	LSC	2000	667	UCU (S) = > UUU (F)	C = > U	1	-	+	-
*rpo*B	3213	+	LSC	2426	809	UCA (S) = > UUA (L)	C = > U	0.86	+	+	U; 5 (25%), C; 15 (75%)
*rpo*C1	2783	+	LSC	41	14	UCA (S) = > UUA (L)	C = > U	1	+	+	U; 5 (27.7%), C; 13 (72.3%)
*rpo*C2	4167	+	LSC	119	40	CCC (P) = > CUC (L)	C = > U	-	+	-	U; 10 (37.1%), C; 17 (62.9%)
*rpo*C2	4167	+	LSC	3731	1244	UCA (S) = > UUA (L)	C = > U	0.86	-	+	-
*rps*2	711	+	LSC	134	45	ACA (T) = > AUA (I)	C = > U	-	+	-	C; 8 (38.1%); U; 13 (61.9%)
*rps*2	711	+	LSC	248	83	UCA (S) = > UUA (L)	C = > U	1	+	+	C; 5 (31.3%), U; 11 (68.7%)
*rps*14	303	+	LSC	80	27	UCA (S) = > UUA (L)	C = > U	1	+	+	C; 5 (5.8%), U; 81 (94.2%)

## Conclusions

Comparison of chloroplast genome organization not only provide us with valuable information for understanding the processes of chloroplast evolution, but also gives insights into the mechanisms underlying genomic rearrangements [25]. Furthermore, investigation of plastid genome structures could trigger further breakthroughs in applied sciences. For example herbicides like PSI and PSII inhibitors have their target genes in the chloroplast genome thus understanding the chloroplast genome may indirectly support the exploration of herbicide resistance and development of novel control methods [89]; while plastid engineering can also be useful to develop resistance to various abiotic and biotic stress factors based on discovered resistance traits. Here we report the complete chloroplast genome sequence of *Solanum dulcamara* as a genomic tool for potential plastid genome comparative studies. We also present the reannotation of Solanaceae plastid genomes using manual curation using *S*. *dulcamara* as a reference. Based on the reannotated genome sequences we introduce a hypothesis of the ancestral plastid genome organization of Solanaceae and the rearrangements unique to some major clades. The ancestral plastid genome of Solanaceae had two degraded non-functional genes, *inf*A and truncated *ycf*1 copy, a deletion in the *trn*A intron and the appearance of a highly divergent gene (*spr*A). Our ancestral genome reconstruction suggests further rearrangements in the stem branch of Solanoideae by the expansion of the IR and the occurrence of a truncated ψ*rps*19 copy at the JLA as a consequence of the expansion. This has been followed by independent rearrangements in deeper nodes such as the accumulation of *trn*F pseudogenes in tandem arrays at a clade referred to as the ‘Pseudosolanoids’ [68] or by the pseudogenization of *spr*A in Physaleae and Capsiceae by two deletions. Further degradation of the *inf*A pseudogene is specific for the largest genus *Solanum*, including tomato and potato.

## Supporting information

S1 Data MAFFT sequence alignment for 35 complete plastid genome sequences used in phylogenetic analysis. (RAR)

S2 Data NEXUS file containing the binary coding used to map genomic changes appearing in the chloroplast genome. (RAR)

S3 Data Annotated checklist of SSRs in *Solanum dulcamara* plastid genome hits founds by MISA. (RAR)

S4 Data Reannotation file of Solanaceae plastid genomes in Geneious format, accessible with 7.1 or later version. (RAR)

S5 Data Reannotation files in GFF and GB file format. (ZIP)

S1 Table NCBI GenBank accession numbers used in this study. (DOCX)

S2 Table List of genes in the chloroplast genome of bittersweet. (DOCX)

S3 Table The genes having intron in the *Solanum dulcamara* plastid genome and the length of the exons and introns. (DOCX)

S4 Table Comparison of major features of *Solanum dulcamara* and nine Solanaceae plastid genomes. (DOCX)

S5 Table Relative synonymous codon usage (RSCU) of *Solanum dulcamara* is given in parentheses following the codon frequency. (DOCX)

S6 Table Estimates of average evolutionary divergence over 80 protein coding-gene sequences from Solanaceae. (DOCX)

S7 Table Total number of perfect simple sequence repeats (SSRs) identified within the chloroplast genome of *Solanum dulcamara*. (DOCX)

S8 Table List of annotation errors found in Solanaceae chloroplast genomes. (XLSX)

S1 Fig Best scoring maximum likelihood trees obtained with RAxML and the most parsimonious tree generated with TNT. (DOCX)

S2 Fig Visualization alignment of chloroplast genome sequences with mVISTA-based identity plots. (PNG)

S3 Fig Alignment of the *spr*A gene in Solanaceae. (PDF)

S4 Fig RNAseq reads mapped to the genomic region of *ycf*15 pseudogene. (PDF)
