# Supplementary material for: The chloroplast genome sequence of bittersweet (Solanum dulcamara): Plastid genome structure evolution in Solanaceae
Source: PLoS One. 2018 Apr 25;13(4):e0196069. doi: 10.1371/journal.pone.0196069 (PMC5919006; doi:10.1371/journal.pone.0196069)
Supplement: S1 Table — (DOCX) [file pone.0196069.s006.docx]

**Table S1** – NCBI GenBank accession numbers used in this study

| **Species** | **Plastid Genome** |  |  |  |  |  |  |
| --- | --- | --- | --- | --- | --- | --- | --- |
| *Atropa bella-donna* | NC004561 |  |  |  |  |  |  |
| *Capsicum annuun* | NC018552 |  |  |  |  |  |  |
| *Capsicum frutescens* | NC028007 |  |  |  |  |  |  |
| *Capsicum lycianthoides* | NC026551 |  |  |  |  |  |  |
| *Datura stramonium* | NC018117 |  |  |  |  |  |  |
| *Dunalia brachycantha* | NC026906 |  |  |  |  |  |  |
| *Dunalia obovata* | NC026563 |  |  |  |  |  |  |
| *Dunalia solanacea* | NC027099 |  |  |  |  |  |  |
| *Hyoscyamus niger* | KF248009 |  |  |  |  |  |  |
| *Iochroma loxense* | NC026726 |  |  |  |  |  |  |
| *Iochroma nitidum* | NC02563 |  |  |  |  |  |  |
| *Iochroma stenanthum* | NC026574 |  |  |  |  |  |  |
| *Iochroma tingoanum* | NC027177 |  |  |  |  |  |  |
| *Nicotiana sylvestris* | NC007500 |  |  |  |  |  |  |
| *Nicotiana tabacum* | NC001879 |  |  |  |  |  |  |
| *Nicotiana tomentosiformis* | NC007602 |  |  |  |  |  |  |
| *Nicotiana undulata* | NC016068 |  |  |  |  |  |  |
| *Physalis peruviana* | NC026570 |  |  |  |  |  |  |
| *Saracha punctata* | NC026694 |  |  |  |  |  |  |
| *Solanum bulbocastanum* | NC007943 |  |  |  |  |  |  |
| *Solanum chilense* | KP117021 |  |  |  |  |  |  |
| *Solanum commersonii* | NC028069 |  |  |  |  |  |  |
| *Solanum dulcamara* | KY863443 |  |  |  |  |  |  |
| *Solanum galapagense* | KP117022 |  |  |  |  |  |  |
| *Solanum habrochaites* | KP117023 |  |  |  |  |  |  |
| *Solanum lycopersicum* | NC007898 |  |  |  |  |  |  |
| *Solanum neorickii* | KP117025 |  |  |  |  |  |  |
| *Solanum nigrum* | NC028070 |  |  |  |  |  |  |
| *Solanum pennelii* | NC024584 |  |  |  |  |  |  |
| *Solanum peruvianum* | KP117026 |  |  |  |  |  |  |
| *Solanum piminellifolium* | KP117027 |  |  |  |  |  |  |
| *Solanum tuberosum* | NC008096 |  |  |  |  |  |  |
| **Outgroups** |  |  |  |  |  |  |  |
| *Coffea arabica* | NC008535 |  |  |  |  |  |  |
| *Ipomoea purpurea* | EU118126 |  |  |  |  |  |  |
| *Ipomoea batatas* | NC026703 |  |  |  |  |  |  |
|  | | |  |  |  |  |  |
