# Supplementary material for: The chloroplast genome sequence of bittersweet (Solanum dulcamara): Plastid genome structure evolution in Solanaceae
Source: PLoS One. 2018 Apr 25;13(4):e0196069. doi: 10.1371/journal.pone.0196069 (PMC5919006; doi:10.1371/journal.pone.0196069)
Supplement: S3 Table — (DOCX) [file pone.0196069.s008.docx]

**Table S3 -** The genes having intron in the *Solanum dulcamara* plastid genome and the length of the exons and introns.

| **Gene** | **Location** | **Exon1 (bp)** | **Intron1 (bp)** | **Exon2 (bp)** | **Intron2 (bp)** | **Exon3 (bp)** |
| --- | --- | --- | --- | --- | --- | --- |
| *rps*16 | LSC | 42 | 856 | 210 |  |  |
| *trn*G-GCC | LSC | 48 | 697 | 23 |  |  |
| *atp*F | LSC | 144 | 691 | 411 |  |  |
| *rpo*C1 | LSC | 435 | 725 | 1623 |  |  |
| *ycf*3 | LSC | 132 | 725 | 228 | 750 | 153 |
| *trn*L-UAA^a^ | LSC | 49 | 497 | 35 |  |  |
| *trn*V-UAC | LSC | 35 | 572 | 45 |  |  |
| *rps*12^b^ | LSC | 114 |  | 232 | 536 | 26 |
| *clp*P | LSC | 71 | 807 | 297 | 625 | 228 |
| *pet*B | LSC | 6 | 750 | 642 |  |  |
| *pet*D | LSC | 8 | 731 | 471 |  |  |
| *rpl*16 | LSC | 9 | 1032 | 396 |  |  |
| *rpl*2 | IR | 393 | 663 | 435 |  |  |
| *ndh*B | IR | 777 | 679 | 756 |  |  |
| *trn*I-GAU | IR | 37 | 727 | 35 |  |  |
| *trn*A-UGC | IR | 38 | 811 | 35 |  |  |
| *ndh*A | SSC | 552 | 1161 | 540 |  |  |

^a^*trn*L-UAA contains the only group I intron, others belong to group II

^b^*rps*12 is a trans-spliced gene with 5' end exon located in the LSC region and the duplicated 3'end exon located in the IR regions.
