# Supplementary material for: The chloroplast genome sequence of bittersweet (Solanum dulcamara): Plastid genome structure evolution in Solanaceae
Source: PLoS One. 2018 Apr 25;13(4):e0196069. doi: 10.1371/journal.pone.0196069 (PMC5919006; doi:10.1371/journal.pone.0196069)
Supplement: S4 Table — (DOCX) [file pone.0196069.s009.docx]

**Table S4** – Comparison of major features of *Solanum dulcamara* and nine Solanaceae plastid genomes

|  | ***Solanum dulcamara*** | ***Solanum tuberosum*** | ***Solanum lycopersicum*** | ***Nicotiana tabacum*** | ***Atropa bella-donna*** | ***Hyosciamus niger*** | ***Capscium annuum*** | ***Datura stramonium*** | ***Physalis peruviana*** | ***Dunalia solanacea*** |
| --- | --- | --- | --- | --- | --- | --- | --- | --- | --- | --- |
| GenBank Nr. | KY863443 | NC008096 | NC007898 | NC001879 | NC004561 | KF248009 | NC018552 | NC018117 | NC026570 | NC027099 |
| Size (bp) | 155,538 | 155,312 | 155,461 | 155,844 | 156,688 | 155,72 | 156,781 | 155,871 | 156,706 | 156,869 |
| LSC length (bp) | 85,901 | 85,749 | 85,876 | 86,684 | 86,868 | 86,105 | 87,366 | 86,302 | 86,996 | 88,743 |
| SSC length (bp) | 18,449 | 18,373 | 18,363 | 18,482 | 18,008 | 17,863 | 25,783 | 18,367 | 18,394 | 18,23 |
| IR length (bp) | 25,615 | 25,595 | 25,611 | 25,339 | 25,906 | 25,876 | 17,849 | 25,602 | 25,658 | 24,948 |
| Total Nr of genes | 112 | 130 | 113 | 122 | 113 | 114 | 113 | 113 | 114 | 112 |
| Nr of genes duplicated in the IR | 18 | 17 | 20 | 24 | 18 | 16 | 20 | 19 | 20 | 20 |
| Nr genes with introns | 18 | 18 | 17 | 15 | 18 | 18 | 17 | 14 | 18 | 16 |
| % GC content | 37,80% | 37% | 37.86% | 40.4% | 37.6% | 37.6% | 37.7% | 37.9% | 37,53% | 37,65% |
