# Supplementary material for: The chloroplast genome sequence of bittersweet (Solanum dulcamara): Plastid genome structure evolution in Solanaceae
Source: PLoS One. 2018 Apr 25;13(4):e0196069. doi: 10.1371/journal.pone.0196069 (PMC5919006; doi:10.1371/journal.pone.0196069)
Supplement: S5 Table — (DOCX) [file pone.0196069.s010.docx]

**Table S5** – Relative synonymous codon usage (RSCU) of *Solanum dulcamara* is given in parentheses following the codon frequency

| **Amino acid** | **Codon** | **No.** | **RSCU** | **Amino acid** | **Codon** | **No.** | **RSCU** |
| --- | --- | --- | --- | --- | --- | --- | --- |
| Gly | GGG | 435 | 1,187 | Trp | UGG | 628 | 1 |
| Gly | GGA | 493 | 1,345 | End | UGA | 826 | 1,265 |
| Gly | GGU | 269 | 0,734 | Cys | UGU | 546 | 1,056 |
| Gly | GGC | 269 | 0,734 | Cys | UGC | 488 | 0,944 |
| Glu | GAG | 230 | 0,557 | End | UAG | 414 | 0,634 |
| Glu | GAA | 596 | 1,443 | End | UAA | 719 | 1,101 |
| Asp | GAU | 498 | 1,46 | Tyr | UAU | 921 | 1,295 |
| Asp | GAC | 184 | 0,54 | Tyr | UAC | 501 | 0,705 |
| Val | GUG | 98 | 0,536 | Leu | UUG | 353 | 1,138 |
| Val | GUA | 130 | 0,711 | Leu | UUA | 434 | 1,399 |
| Val | GUU | 316 | 1,729 | Phe | UUU | 1247 | 1,278 |
| Val | GUC | 187 | 1,023 | Phe | UUC | 705 | 0,722 |
| Ala | GCG | 115 | 0,749 | Ser | UCG | 427 | 0,894 |
| Ala | GCA | 190 | 1,238 | Ser | UCA | 707 | 1,481 |
| Ala | GCU | 163 | 1,062 | Ser | UCU | 682 | 1,428 |
| Ala | GCC | 146 | 0,951 | Ser | UCC | 472 | 0,989 |
| Arg | AGG | 526 | 1,405 | Arg | CGG | 222 | 0,593 |
| Arg | AGA | 806 | 2,152 | Arg | CGA | 358 | 0,956 |
| Ser | AGU | 432 | 0,905 | Arg | CGU | 205 | 0,547 |
| Ser | AGC | 516 | 1,081 | Arg | CGC | 130 | 0,347 |
| Lys | AAG | 401 | 0,59 | Gln | CAG | 153 | 0,481 |
| Lys | AAA | 958 | 1,41 | Gln | CAA | 483 | 1,519 |
| Asn | AAU | 917 | 1,303 | His | CAU | 420 | 1,414 |
| Asn | AAC | 490 | 0,697 | His | CAC | 174 | 0,586 |
| Met | AUG | 238 | 1 | Ser | CUG | 106 | 0,222 |
| Ile | AUA | 305 | 0,53 | Leu | CUA | 162 | 0,522 |
| Ile | AUU | 929 | 1,615 | Leu | CUU | 437 | 1,409 |
| Ile | AUC | 492 | 0,855 | Leu | CUC | 165 | 0,532 |
| Thr | ACG | 273 | 0,822 | Pro | CCG | 137 | 0,702 |
| Thr | ACA | 428 | 1,289 | Pro | CCA | 231 | 1,183 |
| Thr | ACU | 311 | 0,937 | Pro | CCU | 232 | 1,188 |
| Thr | ACC | 316 | 0,952 | Pro | CCC | 181 | 0,927 |
