# Supplementary material for: The chloroplast genome sequence of bittersweet (Solanum dulcamara): Plastid genome structure evolution in Solanaceae
Source: PLoS One. 2018 Apr 25;13(4):e0196069. doi: 10.1371/journal.pone.0196069 (PMC5919006; doi:10.1371/journal.pone.0196069)
Supplement: S6 Table — (DOCX) [file pone.0196069.s011.docx]

**Table** **S6** - Estimates of average evolutionary divergence over 80 protein coding-gene sequences from Solanaceae. Standard error estimate(s) are shown in the fourth column and were obtained by a bootstrap procedure (1,000 replicates). Analyses were conducted using the Kimura 2-parameter model. Codon positions included were 1st + 2nd + 3rd + Noncoding. All positions with less than 95 % site coverage were eliminated. That is, fewer than 5 % alignment gaps, missing data, and ambiguous bases were allowed at any position.

| **Functional Group** | **Gene** | **Location** | **d** | **S.E.** | **Aligned Length** | **Length Min** | **Length Max** |
| --- | --- | --- | --- | --- | --- | --- | --- |
| Ribosomal protein small subunit | *rps*2 | LSC | 0.013 | 0.002 | 711 | 711 | 711 |
|  | *rps*3 | LSC | 0.029 | 0.003 | 657 | 657 | 657 |
|  | *rps*4 | LSC | 0.019 | 0.003 | 609 | 606 | 609 |
|  | *rps*7 | IR | 0.003 | 0.001 | 468 | 468 | 468 |
|  | *rps*8 | LSC | 0.024 | 0.004 | 405 | 405 | 405 |
|  | *rps*11 | LSC | 0.022 | 0.003 | 417 | 417 | 417 |
|  | *rps*12 | IR | 0.003 | 0.001 | 908 | 898 | 908 |
|  | *rps*14 | LSC | 0.019 | 0.003 | 303 | 303 | 303 |
|  | *rps*15 | SSC | 0.031 | 0.005 | 273 | 264 | 273 |
|  | *rps*16 | LSC | 0.040 | 0.003 | 1222 | 1071 | 1137 |
|  | *rps*18 | LSC | 0.016 | 0.003 | 348 | 306 | 342 |
|  | *rps*19 | IR | 0.017 | 0.003 | 279 | 279 | 279 |
| Ribosomal protein large subunit | *rpl*2 | IR | 0.024 | 0.002 | 1528 | 816 | 1491 |
|  | *rpl*14 | LSC | 0.015 | 0.003 | 377 | 370 | 377 |
|  | *rpl*16 | LSC | 0.035 | 0.002 | 1594 | 1364 | 1447 |
|  | *rpl*20 | LSC | 0.039 | 0.005 | 469 | 384 | 419 |
|  | *rpl*22 | LSC | 0.039 | 0.004 | 483 | 468 | 480 |
|  | *rpl*23 | IR | 0.019 | 0.003 | 283 | 265 | 282 |
|  | *rpl*32 | IR | 0.051 | 0.009 | 172 | 158 | 172 |
|  | *rpl*33 | LSC | 0.026 | 0.005 | 216 | 201 | 216 |
|  | *rpl*36 | LSC | 0.029 | 0.009 | 114 | 114 | 114 |
| Subunits of RNA polymerase | *rpo*A | LSC | 0.025 | 0.002 | 1015 | 1014 | 1015 |
|  | *rpo*B | LSC | 0.017 | 0.001 | 3232 | 3204 | 3231 |
|  | *rpo*C1 | LSC | 0.020 | 0.001 | 2920 | 2776 | 2894 |
|  | *rpo*C2 | LSC | 0.026 | 0.001 | 4224 | 4143 | 4194 |
| Photosystem I | *psa*A | LSC | 0.011 | 0.001 | 2253 | 2253 | 2253 |
|  | *psa*B | LSC | 0.012 | 0.001 | 2205 | 2205 | 2205 |
|  | *psa*C | SSC | 0.014 | 0.003 | 246 | 246 | 246 |
|  | *psa*I | LSC | 0.019 | 0.006 | 111 | 105 | 111 |
|  | *psa*J | LSC | 0.012 | 0.004 | 152 | 135 | 152 |
| Photosystem II | *psb*A | LSC | 0.012 | 0.002 | 1062 | 1062 | 1062 |
|  | *psb*B | LSC | 0.011 | 0.001 | 1529 | 1527 | 1529 |
|  | *psb*C | LSC | 0.015 | 0.002 | 1386 | 1386 | 1386 |
|  | *psb*D | LSC | 0.011 | 0.002 | 1062 | 998 | 1062 |
|  | *psb*E | LSC | 0.009 | 0.003 | 252 | 252 | 252 |
|  | *psb*F | LSC | 0.003 | 0.001 | 120 | 120 | 120 |
|  | *psb*H | LSC | 0.028 | 0.007 | 222 | 222 | 222 |
|  | *psb*I | LSC | 0.014 | 0.005 | 119 | 109 | 119 |
|  | *psb*J | LSC | 0.008 | 0.004 | 123 | 123 | 123 |
|  | *psb*K | LSC | 0.013 | 0.003 | 186 | 180 | 186 |
|  | *psb*L | LSC | 0.004 | 0.002 | 117 | 117 | 117 |
|  | *psb*M | LSC | 0.011 | 0.006 | 109 | 98 | 109 |
|  | *psb*N | LSC | 0.007 | 0.002 | 132 | 132 | 132 |
|  | *psb*T | LSC | 0.017 | 0.007 | 105 | 99 | 105 |
|  | *psb*Z | LSC | 0.011 | 0.004 | 189 | 189 | 189 |
| Cytochrome b/f complex | *pet*A | LSC | 0.020 | 0.002 | 963 | 960 | 963 |
|  | *pet*B | LSC | 0.021 | 0.002 | 1494 | 1391 | 1411 |
|  | *pet*D | LSC | 0.026 | 0.002 | 1282 | 1183 | 1242 |
|  | *pet*G | LSC | 0.008 | 0.005 | 114 | 114 | 114 |
|  | *pet*L | LSC | 0.014 | 0.005 | 96 | 96 | 96 |
|  | *pet*N | LSC | 0.014 | 0.006 | 90 | 90 | 90 |
| ATP synthase | *atp*A | LSC | 0.021 | 0.002 | 1524 | 1524 | 1524 |
|  | *atp*B | LSC | 0.018 | 0.002 | 1506 | 1497 | 1500 |
|  | *atp*E | LSC | 0.024 | 0.004 | 407 | 402 | 407 |
|  | *atp*F | LSC | 0.028 | 0.002 | 1346 | 1241 | 1270 |
|  | *atp*H | LSC | 0.013 | 0.003 | 246 | 246 | 246 |
|  | *atp*I | LSC | 0.015 | 0.002 | 744 | 735 | 744 |
| NADH-dehydrogenase | *ndh*A | SSC | 0.040 | 0.002 | 2755 | 2209 | 2539 |
|  | *ndh*B | IR | 0.003 | 0.000 | 2225 | 2198 | 2225 |
|  | *ndh*C | LSC | 0.018 | 0.003 | 363 | 363 | 363 |
|  | *ndh*D | SSC | 0.027 | 0.002 | 1504 | 1503 | 1504 |
|  | *ndh*E | SSC | 0.024 | 0.004 | 306 | 306 | 306 |
|  | *ndh*F | SSC | 0.045 | 0.002 | 2356 | 2175 | 2269 |
|  | *ndh*G | SSC | 0.025 | 0.003 | 531 | 530 | 531 |
|  | *ndh*H | SSC | 0.026 | 0.002 | 1188 | 1182 | 1188 |
|  | *ndh*I | SSC | 0.022 | 0.003 | 505 | 504 | 505 |
|  | *ndh*J | LSC | 0.019 | 0.003 | 498 | 477 | 498 |
|  | *ndh*K | LSC | 0.021 | 0.002 | 696 | 672 | 690 |
| Large subunit Rubisco | *rbc*L | LSC | 0.021 | 0.002 | 1434 | 1434 | 1434 |
| Acetyl-CoA carboxylase | *acc*D | LSC | 0.029 | 0.002 | 1861 | 1515 | 1656 |
| Cytochrome c biogenesis | *ccs*A | SSC | 0.040 | 0.003 | 993 | 939 | 969 |
| Maturase | *mat*K | LSC | 0.042 | 0.003 | 1613 | 1544 | 1544 |
| ATP-dependent protease | *clp*P | LSC | 0.058 | 0.003 | 2211 | 1988 | 2029 |
| Inner membrane protein | *cem*A | LSC | 0.066 | 0.006 | 691 | 690 | 691 |
| Conserved hypothetical chloroplast ORF | *ycf*1 | SSC | 0.021 | 0.002 | 6794 | 5622 | 6174 |
|  | *ycf*2 | IR | 0.012 | 0.001 | 7195 | 6622 | 6894 |
|  | *ycf*3 | LSC | 0.021 | 0.002 | 2124 | 1958 | 2034 |
|  | *ycf*4 | LSC | 0.022 | 0.003 | 556 | 556 | 556 |
|  | *ycf*15 | IR | 0.007 | 0.002 | 277 | 264 | 277 |
|  | ***spr*A** | SSC | **0.114** | 0.016 | 311 | 140 | 256 |
|  |  |  |  |  |  |  |  |
