# Supplementary material for: The chloroplast genome sequence of bittersweet (Solanum dulcamara): Plastid genome structure evolution in Solanaceae
Source: PLoS One. 2018 Apr 25;13(4):e0196069. doi: 10.1371/journal.pone.0196069 (PMC5919006; doi:10.1371/journal.pone.0196069)
Supplement: S7 Table — (DOCX) [file pone.0196069.s012.docx]

**Table S7 -** Total number of perfect simple sequence repeats (SSRs) identified within the chloroplast genome of *Solanum dulcamara*

| **Repeats** | **3** | **4** | **5** | **6** | **7** | **8** | **9** | **10** | **11** | **12** | **13** | **14** | **Total** |
| --- | --- | --- | --- | --- | --- | --- | --- | --- | --- | --- | --- | --- | --- |
| A/T | - | - | - | - | 128 | 43 | 28 | 13 | 14 | 4 | 3 | 4 | 237 |
| C/G | - | - | - | - | 15 | 1 |  |  |  |  |  |  | 16 |
| AG/CT | - | 16 |  |  |  |  |  |  |  |  |  |  | 16 |
| AT/AT | - | 15 | 8 |  | 1 |  |  |  |  |  |  |  | 24 |
| AAC/GTT | 9 |  |  |  |  |  |  |  |  |  |  |  | 9 |
| AAG/CTT | 20 |  |  |  |  |  |  |  |  |  |  |  | 20 |
| AAT/ATT | 21 | 1 | 1 |  |  |  |  |  |  |  |  |  | 23 |
| ACC/GGT | 1 |  |  |  |  |  |  |  |  |  |  |  | 1 |
| ACG/CGT | 1 |  |  |  |  |  |  |  |  |  |  |  | 1 |
| ACT/AGT | 1 |  |  |  |  |  |  |  |  |  |  |  | 1 |
| AGC/CTG | 6 |  |  |  |  |  |  |  |  |  |  |  | 6 |
| AGG/CCT | 4 |  |  |  |  |  |  |  |  |  |  |  | 4 |
| ATC/ATG | 5 |  |  |  |  |  |  |  |  |  |  |  | 5 |
| AAAC/GTTT | 3 |  |  |  |  |  |  |  |  |  |  |  | 3 |
| AAAT/ATTT | 4 |  |  |  |  |  |  |  |  |  |  |  | 4 |
| AATT/AATT | 1 |  |  |  |  |  |  |  |  |  |  |  | 1 |
| AGAT/ATCT | 2 |  |  |  |  |  |  |  |  |  |  |  | 2 |
| AATTC/AATTG | 1 |  |  |  |  |  |  |  |  |  |  |  | 1 |
