# Supplementary material for: The chloroplast genome sequence of bittersweet (Solanum dulcamara): Plastid genome structure evolution in Solanaceae
Source: PLoS One. 2018 Apr 25;13(4):e0196069. doi: 10.1371/journal.pone.0196069 (PMC5919006; doi:10.1371/journal.pone.0196069)
Supplement: S1 Fig — (DOCX) [file pone.0196069.s014.docx]

**Figure X**. The best maximum-likelihood (ML) trees inferred from the plastid genome dataset. Each tree is visualized as cladogram with statistical node support and as phylogram with exact branch lengths. Bootstrap support values of the ML inference greater than 50% are given above branches in the cladograms. **(a)** Tree topology inferred under an unpartitioned whole plastid genome alignment with one of the IRs removed; **(b)** tree topology for the same data matrix but partitioning carried out by gene, exon, intron and intergenic spacer regions (n = 258); **(c)** tree topology for the full plastid genome alignment with partitions inferred with PartitionFinder2. **(d)** Tree topology inferred with parsimony using TNT. Bootstrap support values were inferred from 1000 replications and represent 100% for support for all nodes.

**Best ML tree as cladogram** **Best ML tree as phylogram**

1.
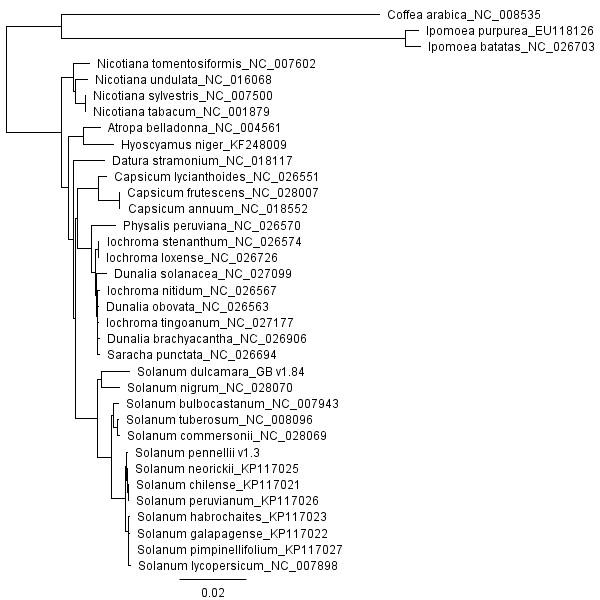

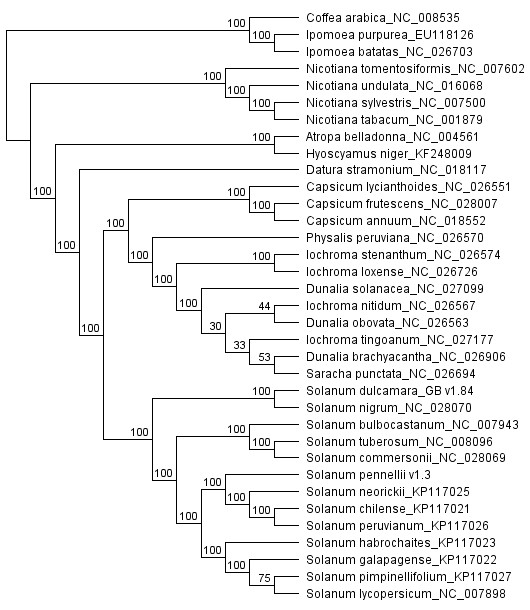


**
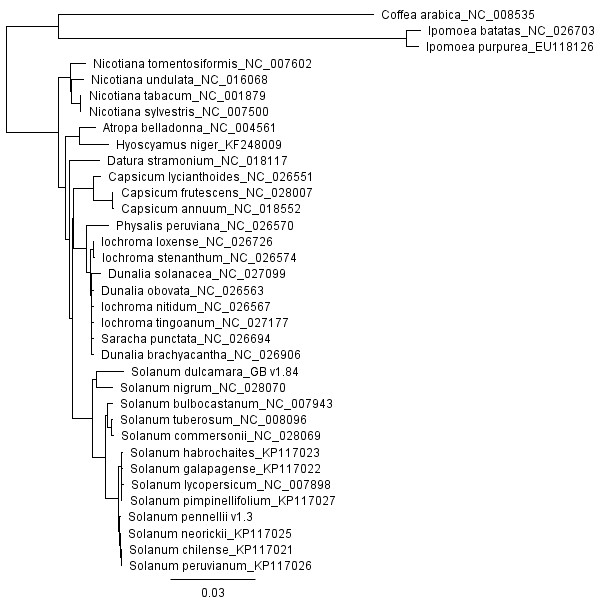
**
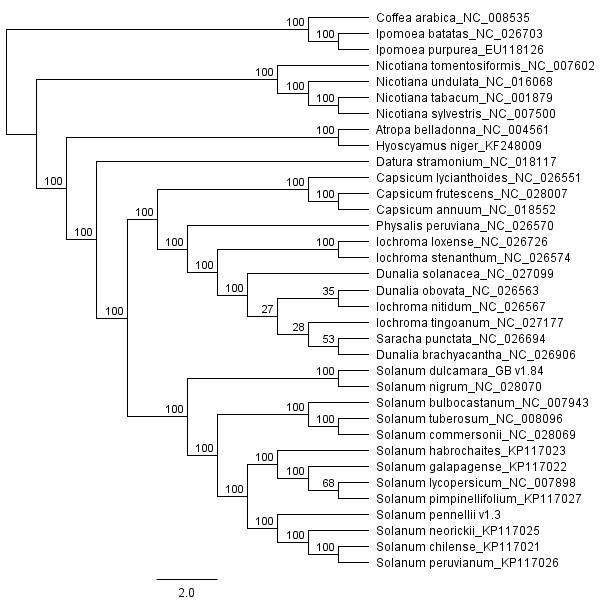
**(b)**

**(c)**

**
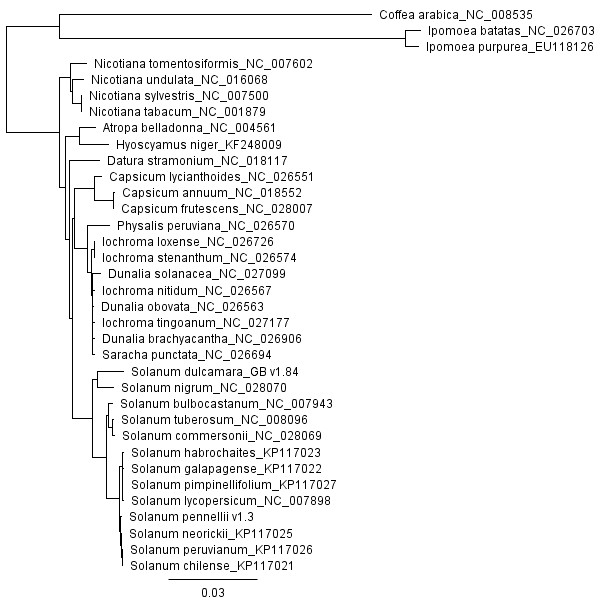
**
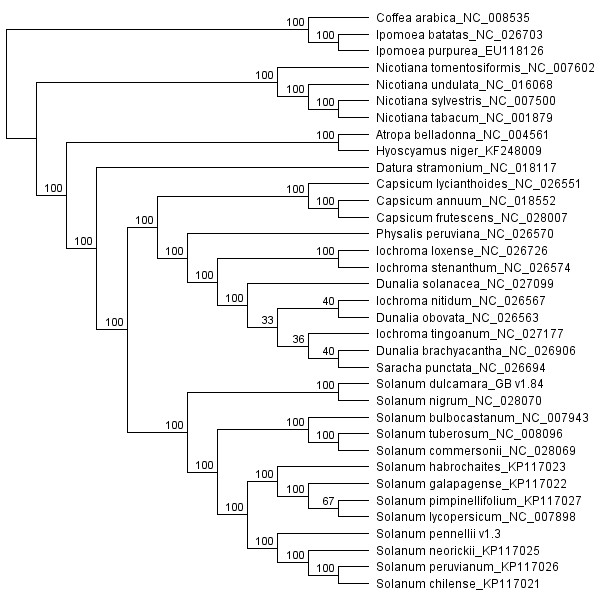


**(d)**

**
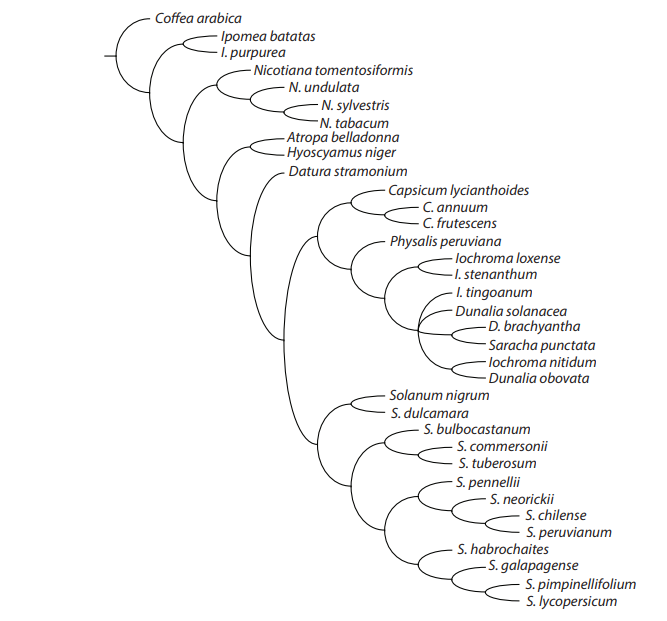
**
