# Supplementary figures and images for: The chloroplast genome sequence of bittersweet (Solanum dulcamara): Plastid genome structure evolution in Solanaceae
Source: PLoS One. 2018 Apr 25;13(4):e0196069. doi: 10.1371/journal.pone.0196069 (PMC5919006; doi:10.1371/journal.pone.0196069)

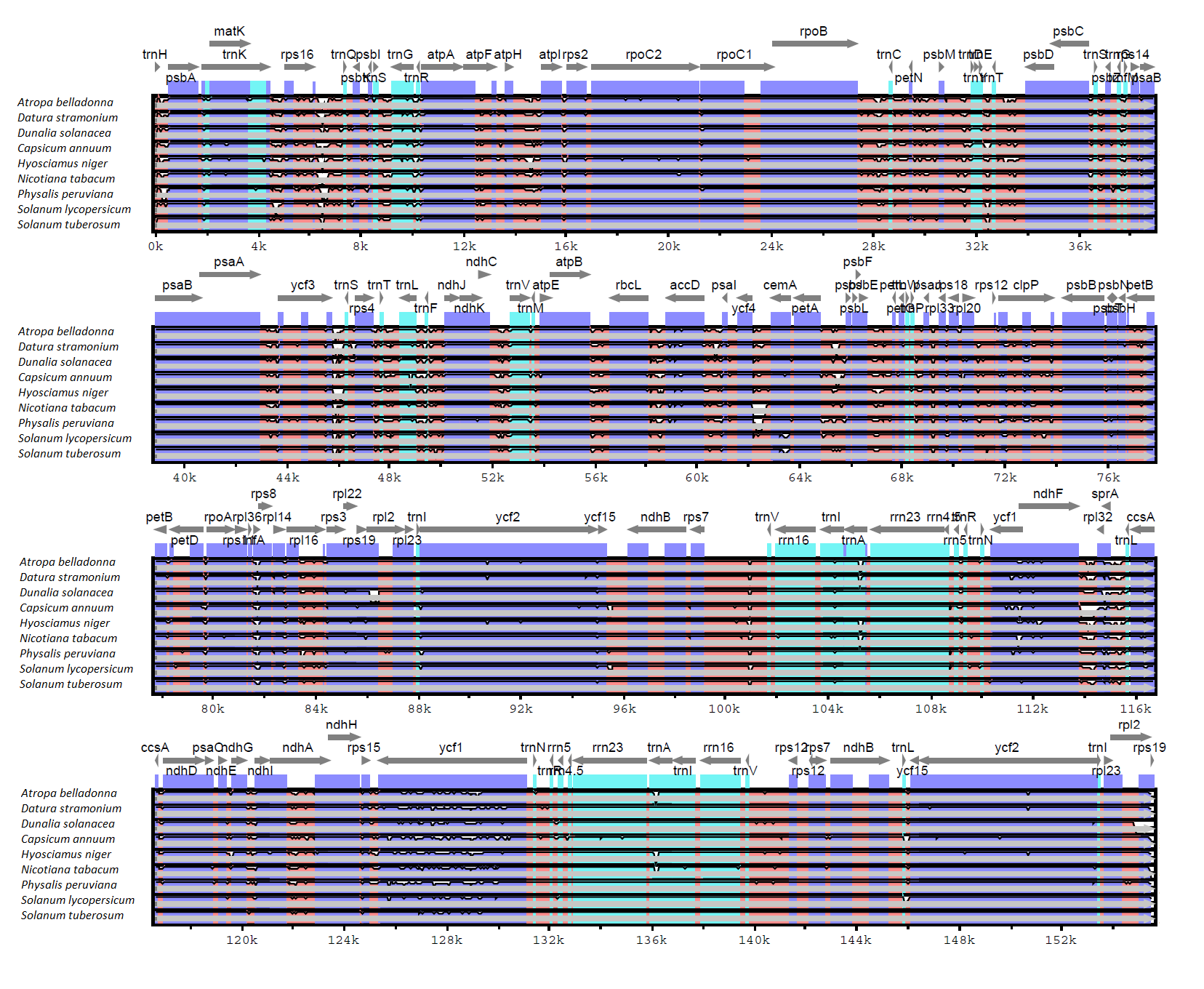

Supplement: S2 Fig — (PNG) [file pone.0196069.s015.png]

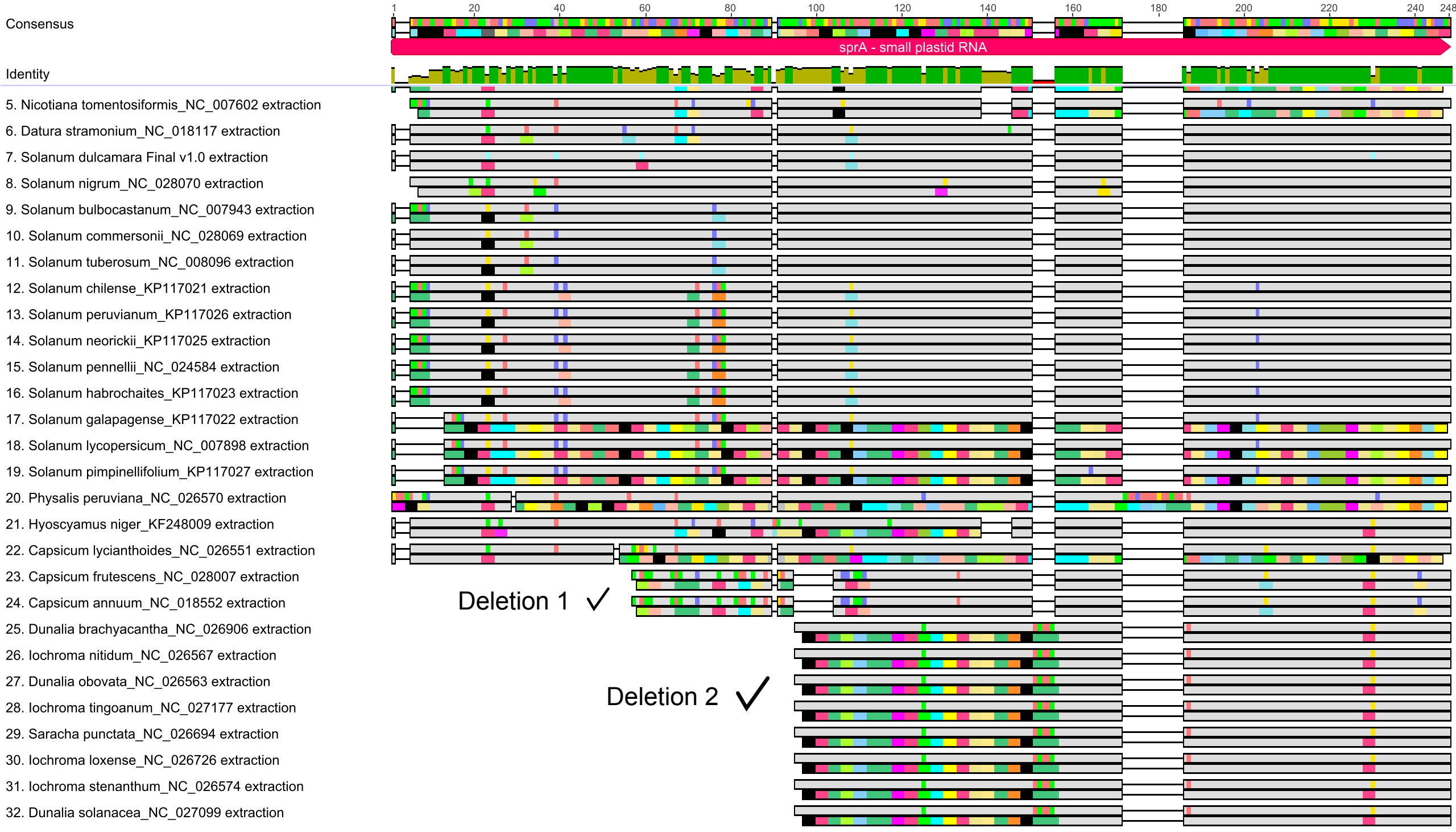

Supplement: S3 Fig — (PDF) [file pone.0196069.s016.pdf]

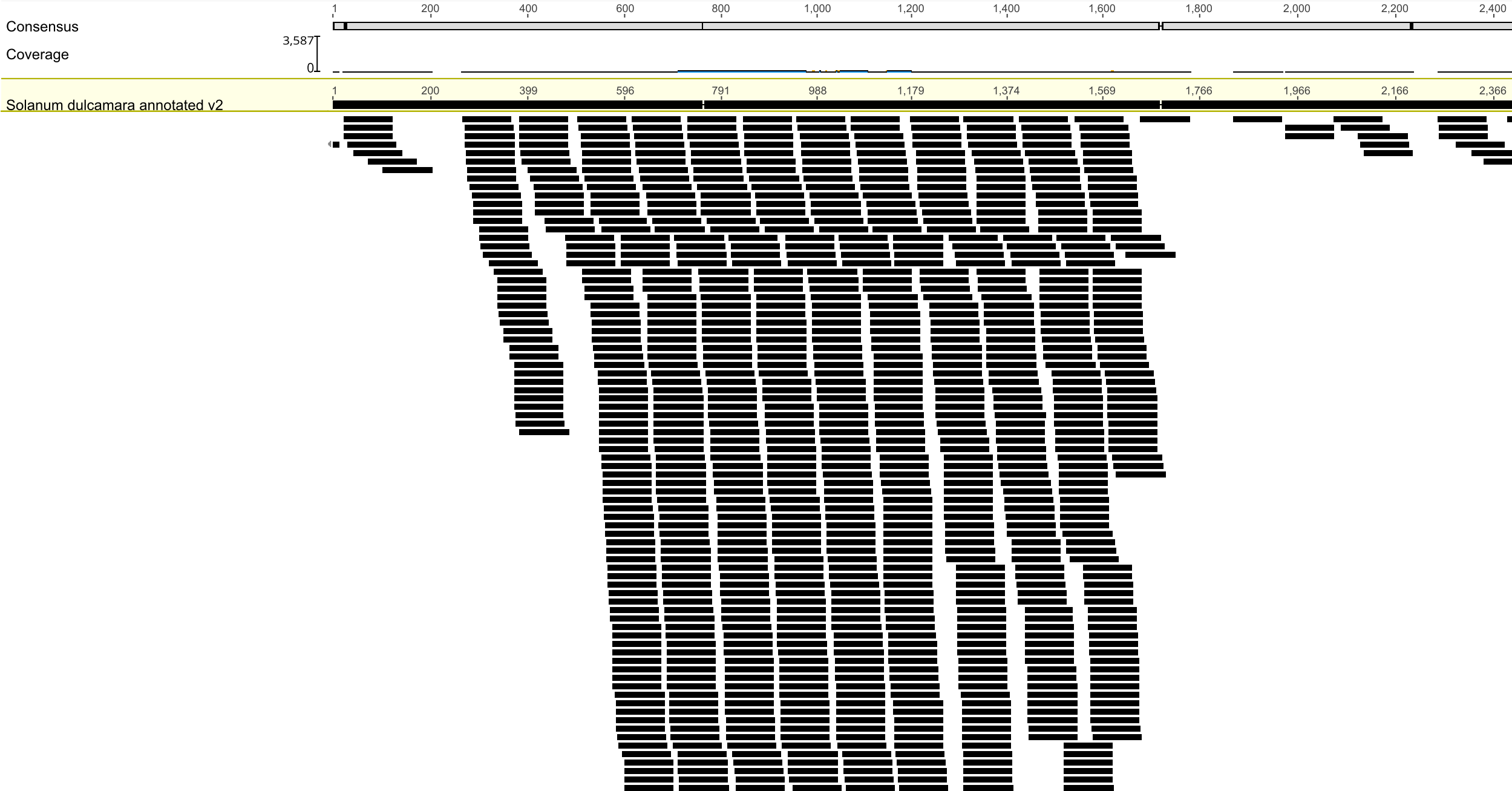

Supplement: S4 Fig — (PDF) [file pone.0196069.s017.pdf]
